# The *Aspergillus nidulans* Zn(II)_2_Cys_6_ transcription factor AN5673/RhaR mediates L-rhamnose utilization and the production of α-L-rhamnosidases

**DOI:** 10.1186/s12934-014-0161-9

**Published:** 2014-11-22

**Authors:** Ester Pardo, Margarita Orejas

**Affiliations:** Instituto de Agroquímica y Tecnología de Alimentos, Consejo Superior de Investigaciones Científicas (IATA-CSIC), Agustín Escardino 7, 46980 Paterna, Valencia Spain

**Keywords:** α-L-rhamnosidases, *Aspergillus nidulans*, AN5673/*rhaR*, L-rhamnose metabolic pathway, *LRA* gene cluster, AN5672/*lraC*, *Neurospora crassa*, NCU9033/*rhaR*, non-radioactive EMSA, TF knockout

## Abstract

**Background:**

Various plant-derived substrates contain L-rhamnose that can be assimilated by many fungi and its liberation is catalyzed by α-L-rhamnosidases. Initial data obtained in our laboratory focussing on two *Aspergillus nidulans* α-L-rhamnosidase genes (*rhaA* and *rhaE*) showed α-L-rhamnosidase production to be tightly controlled at the level of transcription by the carbon source available. Whilst induction is effected by L-rhamnose, unlike many other glycosyl hydrolase genes repression by glucose and other carbon sources occurs in a manner independent of CreA. To date regulatory genes affecting L-rhamnose utilization and the production of enzymes that yield L-rhamnose as a product have not been identified in *A. nidulans*. The purpose of the present study is to characterize the corresponding α-L-rhamnosidase transactivator.

**Results:**

In this study we have identified the *rhaR* gene in *A. nidulans* and *Neurospora crassa* (AN5673, NCU9033) encoding a putative Zn(II)_2_Cys_6_ DNA-binding protein. Genetic evidence indicates that its product acts in a positive manner to induce transcription of the *A. nidulans* L-rhamnose regulon. *rhaR*-deleted mutants showed reduced ability to induce expression of the α-L-rhamnosidase genes *rhaA* and *rhaE* and concomitant reduction in α-L-rhamnosidase production. The *rhaR* deletion phenotype also results in a significant reduction in growth on L-rhamnose that correlates with reduced expression of the L-rhamnonate dehydratase catabolic gene *lraC* (AN5672). Gel mobility shift assays revealed RhaR to be a DNA binding protein recognizing a partially symmetrical CGG-X_11_-CCG sequence within the *rhaA* promoter. Expression of *rhaR* alone is insufficient for induction since its mRNA accumulates even in the absence of L-rhamnose, therefore the presence of both functional RhaR and L-rhamnose are absolutely required. In *N. crassa*, deletion of *rhaR* also impairs growth on L-rhamnose.

**Conclusions:**

To define key elements of the L-rhamnose regulatory circuit, we characterized a DNA-binding Zn(II)_2_Cys_6_ transcription factor (RhaR) that regulates L-rhamnose induction of α-L-rhamnosidases and the pathway for its catabolism in *A. nidulans*, thus extending the list of fungal regulators of genes encoding plant cell wall polysaccharide degrading enzymes. These findings can be expected to provide valuable information for modulating α-L-rhamnosidase production and L-rhamnose utilization in fungi and could eventually have implications in fungal pathogenesis and pectin biotechnology.

**Electronic supplementary material:**

The online version of this article (doi:10.1186/s12934-014-0161-9) contains supplementary material, which is available to authorized users.

## Background

Saprophytic and pathogenic fungi hierarchically utilize plant biomass components as sources of carbon and energy. L-Rhamnose is a naturally occurring deoxyhexose sugar (6-deoxy-L-mannose) that can be assimilated by numerous yeasts and filamentous fungi when preferred carbon sources such as glucose are limited or absent. L-Rhamnose is widely distributed in plants where it is commonly found in glycosyl linkage to other sugars and organic moieties including the primary cell wall pectic polysaccharides rhamnogalacturonan I and II, hemicellulose, glycoproteins and diverse secondary metabolites, some of the latter being important bioactive compounds (*e.g.* carotenoids, flavonoids, saponins, glycopeptide antibiotics, anthocyanins and glycoalkaloids - see [1] and references therein). L-Rhamnose released from the degradation of these plant materials induces the production of various enzymes appropriate for the continued depolymerisation/modification and utilization of these substrates.

L-Rhamnose and rhamnosides are promising candidates for use in the fields of food, cosmetics, agriculture and health, and improvement of the physicochemical properties of a number of L-rhamnose containing phytochemicals upon removal of the rhamnose moiety has been shown [1]. Thus, α-L-rhamnosidases (CAZy GH families 13, 78, 106 and possibly 28) - enzymes which specifically catalyse the hydrolysis of terminal non-reducing L-rhamnose residues in oligosaccharides and α-L-rhamnosides - find a variety of uses in industry that include the reduction of citrus juice bitterness, the improvement of aroma release in musts and wines, increasing the bioavailability of food ingredients, the production of sweetener precursors, the biotransformation of antibiotics, flavonoids and steroids (*i.e.* drug development), and the production of L-rhamnose, as well as applications in the bioethanol industry, etc. [1-5 and references therein]. In this regard, engineered fungal cells with enhanced rhamnosidase yields have been reported [6,7]. In addition, these enzymes also are involved in the detoxification of plant secondary metabolites and hence they could play a role in evading plant defences against fungal attacks [8,9 and references therein]. Thus, in some instances optimizing the biosynthesis of α-L-rhamnosidases is useful whereas in other situations abolishing it is the preferable option. To this end, detailed knowledge of the regulatory mechanisms underlying their production will help towards engineering improved fungal strains and/or process conditions.

In *Aspergillus nidulans,* α-L-rhamnosidase production is tightly controlled by the available carbon source [10,11]. Focussing on two *A. nidulans* α-L-rhamnosidase genes (*rhaA* and *rhaE*) we have shown that this control occurs at the level of transcription, being induced by L-rhamnose but, unlike many other glycosyl hydrolase genes, being repressed by glucose and other carbon sources in a manner independent of CreA, the only carbon catabolite repressor protein known to date in filamentous fungi. In addition, studies on mixed carbon sources indicate that inducer exclusion may play a prominent role in α-L-rhamnosidase gene regulation [11]. However, regulatory genes affecting L-rhamnose utilization and the production of enzymes that yield L-rhamnose as a product have not been identified in *A. nidulans*. The purpose of the present study is to characterize the corresponding *A. nidulans* α-L-rhamnosidase transactivator.

**Figure 1:**
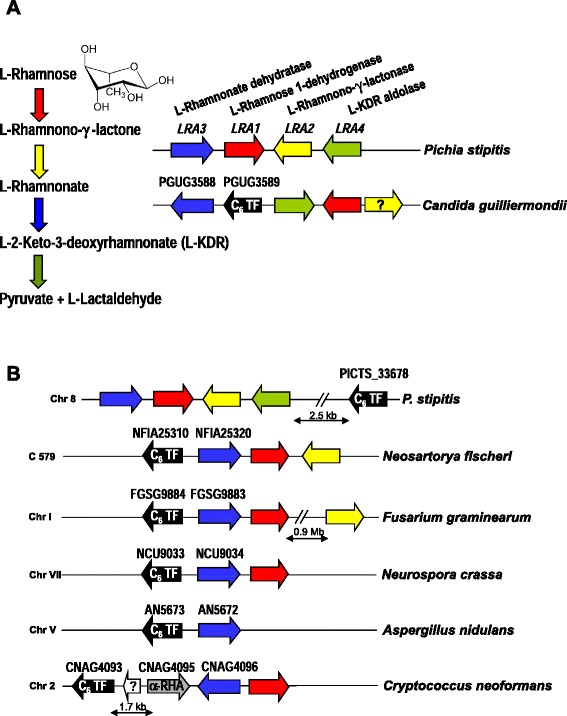
**Schematic fungal L-rhamnose (** ***LRA*** **) gene clusters. (A)** Catabolic pathway for L-rhamnose and the gene cluster encoding the corresponding activities in *P. stipitis* and *C. guilliermondii* (according to [15]). **(B)** Location of the hypothetical pathway-specific regulatory gene *rhaR* (black arrow) in relation to the proposed *LRA* catabolic genes in yeast and filamentous fungi. The arrows indicate the direction of transcription based on sequence analyses. Homologous genes are indicated using the same colour code. Chr and C indicate chromosome number and contig respectively; α-RHA indicates α-L-rhamnosidase.

Enzymes involved in the sequential steps of specific fungal metabolic pathways are often encoded by sets of contiguous genes that are co-ordinately regulated (*i.e.* gene clusters). Many pathway-specific regulatory proteins contain a Zn(II)_2_Cys_6_ DNA binding domain [12,13], the hallmark of this class of fungal transcription factors, and are encoded by a gene often associated with these metabolic gene clusters [14]. Thus, once the gene for a catabolic enzyme is identified, there may be good possibility to locate candidate sequences encoding pathway specific regulators. Such is the case for the L-rhamnose catabolic pathway (encoded by the *LRA* cluster) that has recently been characterized in *Scheffersomyces* (*Pichia*) *stipitis* (Figure 1A) and comprises the enzymes L-rhamnose dehydrogenase (encoded by *LRA1/RHA1*), L-rhamnono-1,4-lactonase (*LRA2*), L-rhamnonate dehydratase (*LRA3*) and L-KDR aldolase (*LRA4*) which yield the degradation products pyruvate and L-lactaldehyde [15]. Bioinformatic analysis of a number of fungal genomes showed partial conservation of this genomic architecture. Interestingly, a gene (PGUG_03589) encoding a Zn(II)_2_Cys_6_ protein was found to be located between the *LRA3* and *LRA4* homologues in *Candida guilliermondii* [15], suggesting the possibility of a regulatory role for its product in controlling nearby genes.

As a means to study the mechanism of L-rhamnose induction in *A. nidulans* we have characterized the specific transactivator (RhaR) of the *rhaA*, *rhaE* and *lraC* (*vide infra*) genes, thus extending the list of fungal regulators of genes encoding plant cell wall polysaccharide degrading enzymes. These findings can be expected to provide valuable information for modulating α-L-rhamnosidase production and L-rhamnose utilization in fungi and could eventually have implications in fungal pathogenesis and pectin biotechnology.

## Results and discussion

### Identification of the *A. nidulans* putative α-L-rhamnosidase transactivator based on conspicuous genomic associations

From earlier *in silico* analyses (our unpublished data) we found that genes homologous to PGUG_03589 (encoding a putative Zn(II)_2_Cys_6_ regulatory protein within the *LRA* cluster; [15]) in diverse fungi, including *P. stipitis*, are also consistently linked to the hypothetical L-rhamnose catabolic genes (Figure 1B and results recently published [16] while this work was in preparation), and whilst their function is as yet unknown they could be involved in regulating nearby genes. Mining the *A. nidulans* genome we found that the gene homologous to PGUG_03589 (locus AN5673; currently annotated as a possible pseudogene) also maps tightly linked to the homologue of the *P. stipitis LRA3* (AN5672/*lraC*), making AN5673 a candidate for encoding a pathway-specific transcriptional factor (hereafter termed *rhaR*) controlling the L-rhamnose regulon.

**Figure 2:**
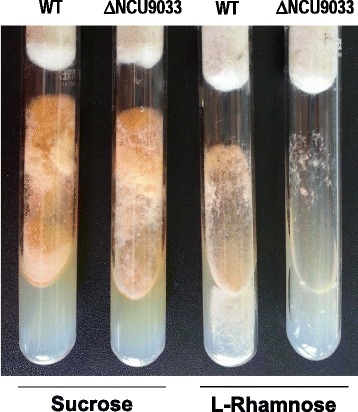
**Impaired growth on L-rhamnose of the** ***N. crassa*** **transcription factor NCU9033 knockout mutant.** Wild type (FGSC 2489) and ∆NCU9033 (FGSC 11390) strains were grown on Vogel’s minimal media (with 55 mM of sucrose or L-rhamnose as the sole carbon sources) slant tubes at 30°C for 5 days and then at room temperature for 3 days (daylight) to analyze hyphae production and pigmentation. Equal numbers of conidia (3000) were inoculated.

The availability of a collection of *Neurospora crassa* transcription factor deletion strains [17] enabled us to carry out a preliminary study in this fungus oriented towards testing that hypothesis. In this regard, we assessed whether the *N. crassa* mutant deleted for the *rhaR* homologue (∆NCU09033; FGSC 11390; [18]) was able to grow on L-rhamnose as a sole carbon source. As shown in Figure 2, the ∆NCU09033 strain exhibited poor growth on this carbon source compared to the wild type reference strain 74*A* (FGSC 2489) whereas growth on sucrose-containing medium was unaffected. This strongly suggests that the deleted gene plays a role in the regulatory circuit that controls L-rhamnose utilization in *N. crassa* and by extension in other fungi. These observations led us to characterize the role of *rhaR* in *A. nidulans*.

### Gene structure of *A. nidulans rhaR*

The *A. nidulans rhaR* (rhamnosidase regulator) gene is 2800 bp in length and maps to chromosome V. It is divergently transcribed from *lraC* and their predicted translation start sites are separated by 742 bp. Synteny between these two genes also occurs in a number of filamentous ascomycetes including those species for which putative gene clusters related to L-rhamnose metabolism were first identified ([15]; also see Figure 1B). Using RT-PCR, cDNA fragments corresponding to the ORF of the gene were generated and sequenced. Comparison of the cDNA and genomic sequences (Additional file 1: Figure S1A) revealed the presence of four introns of 154, 50, 51 and 52 nucleotides which conform to the canonical GT-AG splicing rule [19]. The positions of the third and fourth introns (L111/D112 - 21 amino acids after the Zn(II)_2_Cys_6_ region, and Q568/T569) are conserved in the homologous *Cryptococcus neoformans* gene, suggesting that these introns were present in the last common dikarya ancestor. AN5673 is misannotated in both the Broad and AspGD databases due to failure to recognize introns in the Cys_6_ encoding region.

The predicted polypeptide encoded by the *A. nidulans rhaR* gene is 830 amino acids (aa) long (Additional file 1: Figure S1A) and has a calculated molecular mass of 92.6 kDa. The Zn(II)_2_Cys_6_ binuclear cluster domain is found near the N-terminus (residues 62–89; C-X_2_-C-X_6_-C-X_6_-C-X_2_-C-X_6_-C), and the second intron interrupts the coding region of this sequence between the third and the fourth cysteine residues. Like other zinc binuclear cluster proteins [20-22], RhaR contains one putative dimerization coiled-coil domain C-terminal to the Cys_6_ region (residues G102-T127; PEPCOIL score 2.38, probability 1 using a window of 14 residues), suggesting that RhaR may bind as a dimer to roughly symmetrical DNA target sites containing a conserved trinucleotide sequence (usually CGG) at either end. In this regard, pioneering work carried out on the zinc binuclear cluster proteins Gal4 and Ppr1 of *S. cerevisiae* established that two lysine residues between the second and the third cysteines (K17 and K18 in Gal4 – Additional file 1: Figure S1B) make all the specific base contacts to the CGG repeats whereas the linker region between the Zn(II)_2_Cys_6_ and the coiled-coil domains specifies the length of the sequence between the triplets [13,23]. K18 is conserved in RhaR (K69) whereas K17 is replaced by arginine (R68). Other proteins with this substitution also seem to recognize CGG, whilst the binding sites of some proteins of this family of transcription regulators differ - different terminal triplets as well as alternate asymmetrical sites have been described - [20-22]. The conserved proline residue positioned between the third and fourth cysteines which is important in Gal4 to avoid strain in the loop formed between these cysteines [13] is also present in RhaR (P77). A truncated fungal-specific transcription factor domain ([20]; PF04082; residues V263-E533) which includes the middle homology region (MHR) is located in the middle of the protein sequence (Additional file 1: Figure S1C), and a putative importin α-dependent bipartite nuclear localization signal (cNLS score >9; http//nls-mapper.iab.keio.ac.jp/; [24]) is also present that extends from R50 to K71 and partially overlaps the proposed DNA binding region, further supporting the possibility of a role for RhaR as a transcriptional regulator.

### Deletion of *rhaR* severely impairs α-L-rhamnosidase activity

To gain insight into the role of the *A. nidulans rhaR* gene product a targeted disruption of the gene was carried out in a ∆*nkuA* host strain (AR198) to facilitate the desired homologous recombination [25]. Riboflavin prototrophic transformants (T1-T12) were selected on media (MM-glucose) lacking riboflavin and then replica-plated onto inducing media (MM-rhamnose) supplemented with the artificial substrate MUR to screen for colonies deficient in α-L-rhamnosidase activity. In contrast to the untransformed strain and the riboflavin nutritional control AR271 (AR198 complemented with the *riboB* cassette of *Aspergillus fumigatus*; see Methods), no activity against MUR was found in any of the twelve transformants (Additional file 2: Figure S2A) strongly suggesting that they resulted from homologous integration and gene replacement (*rhaR*::Af*riboB*). This is consistent with an important positive role for *rhaR* in the biosynthesis of α-L-rhamnosidases in *A. nidulans*. Diagnostic PCR (using primer pairs that confirm the presence of the correctly integrated deletion cassette and the parental *rhaR* gene) (Additional file 2: Figure S2B) and Southern analysis (not shown) were used to verify *rhaR* gene deletion (Δ*rhaR*) in two randomly selected transformants, T4 (AR225) and T11 (AR227) that were chosen for further work.

In order to avoid the possible influence of pleiotropic effects of the ∆*nkuA* allele on the ∆*rhaR* phenotype and random mutations introduced during the transformation process, sexual crosses were performed to recover ∆*rhaR nkuA*^*+*^ strains. Transformant AR225 (relevant genotype: *argB2,* ∆*nkuA*::Af*argB, pyroA4,* ∆*rhaR::*Af*riboB*) was crossed to AR4 (*argB2, metG1, biA1*) and the progeny were plated on MM-glucose supplemented with arginine but lacking riboflavin. The absence of α-L-rhamnosidase activity on MM-rhamnose-MUR plates in two randomly selected riboflavin prototrophic and arginine auxotrophic descendants (AR234 and AR237) strongly supports a direct relationship between the ∆*rhaR* allele and the absence of α-L-rhamnosidase activity (Additional file 2: Figure S2C).

### Re-introduction of *rhaR* into ∆*rhaR* mutant strains restores α-L-rhamnosidase activity

To confirm that the Δ*rhaR* mutant phenotype was the result of *rhaR* deletion alone, complementation of the deletion was undertaken with the wild type *rhaR* gene subcloned into pILJ16 (carries the *argB* gene - [26]) and the resulting plasmid (pILJ16_rhaR) was transformed into the ∆*rhaR* mutant AR237 (relevant genotype ∆*rhaR::*Af*riboB, nkuA*^+^*, argB2*). Since integration of DNA in strains carrying the wild type *nkuA* allele is predominantly ectopic this dramatically reduces the likelihood of recombination at the homologous locus. Of forty-seven arginine prototrophic transformants that were selected and grown on medium containing L-rhamnose and the fluorescent substrate MUR (Rha-MUR plates), 17 were able to degrade MUR (Additional file 2: Figure S2D). Thus, the *rhaR* gene was able to restore the rhaR^+^ phenotype to the *A. nidulans* ∆*rhaR* strain as manifested by the production of α-L-rhamnosidase activity. PCR analyses confirmed the unaltered nature of the ∆*rhaR::*Af*riboB* structure at the *rhaR* locus (Additional file 2: Figure S2B) and hence ectopic reinsertion of *rhaR* in two selected transformants (AR256 and AR262). Taken together with the previous data this confirms a role for the *A. nidulans rhaR* gene in the production of α-L-rhamnosidase activity.

### RhaR controls L-rhamnose induction of α-L-rhamnosidase biosynthesis at the transcriptional level

**Figure 3:**
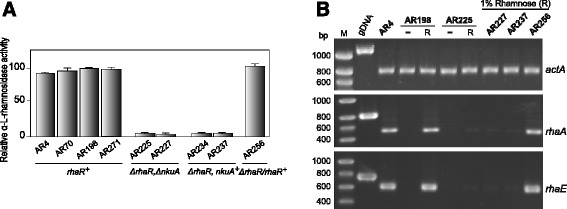
**Extracellular α-L-rhamnosidase activity and α-L-rhamnosidase gene expression in** ***rhaR*** ^**+**^ **and ∆** ***rhaR*** **strains. (A)** Bar diagram of the relative α-L-rhamnosidase activities secreted by *rhaR* ^+^ (AR4, AR70, AR198, AR271 and AR256) and ∆*rhaR* (AR225, AR227, AR237 and AR234) strains after 48 h grown in 1% w/v L-rhamnose. Activities are presented as percentages of those observed in AR198, and values are presented as the mean of at least three independent experiments and their standard deviation. **(B)** RT-PCR analyses for *rhaA* and *rhaE* in *rhaR* ^+^ and ∆*rhaR* strains under inducing conditions using RNAs isolated from mycelia obtained after 4 h transfer to 1% w/v L-rhamnose. The actin *actA* gene (AN6542) was used as a constitutive control for normalization. RNA quality and amount was also verified by ethidium bromide staining of rRNAs (not shown).

To confirm the qualitative results obtained on MUR plates (Additional file 2: Figure S2), Δ*rhaR* transformants were analysed for their ability to synthesize α-L-rhamnosidase activities after 48 h of growth in YMM liquid media containing 1% L-rhamnose as the sole carbon source. Enzyme activities were measured using *p*NPR as substrate. In contrast to *rhaR*^+^ strains (*i.e.* untransformed AR198, the parental AR4, the arginine nutritional control AR70, the riboflavin nutritional control AR271 and the ∆*rhaR* complemented AR256), α-L-rhamnosidase activity was severely reduced (by about 95%) in the two selected Δ*rhaR* mutants (AR225 and AR227) as well as in two Δ*rhaR* descendants of AR225 (AR234 and AR237) (Figure 3A), demonstrating that the *A. nidulans rhaR* gene is necessary for the synthesis of α-L-rhamnosidases. Bearing in mind that the product of *rhaR* is a Zn(II)_2_Cys_6_ protein, these results suggest that this could be the consequence of reduced expression of α-L-rhamnosidase encoding genes.

Previous work in *A. nidulans* has shown that the transcription of two functionally characterized α-L-rhamnosidase genes (*rhaA* and *rhaE*) the products of which exhibit different substrate specificities - pectic rhamnogalacturonan oligomers and flavonoids, respectively [27,28] - is induced by L-rhamnose [11]. To assess whether RhaR functions as a transcriptional regulator, the steady-state levels of *rhaA* and *rhaE* mRNAs in AR225, AR227 and AR237 (∆*rhaR* strains) were compared to those in selected *rhaR*^*+*^ controls (AR198, AR4 and AR256) by semiquantitative RT-PCR. Total RNAs were isolated from 18 h D-fructose (0.1%) grown mycelia transferred to rhamnose (1%) medium for 4 h. Consistent with the activity data, Figure 3B shows that in the presence of L-rhamnose (inducing conditions) the expression of *rhaA* and *rhaE* is greatly reduced in the gene deletion strains compared to the controls whilst expression of the reference gene *actA* was similar in both, clearly indicating that the product of *rhaR* plays a positive role in α-L-rhamnosidase production at the transcriptional level. RT-PCR analysis also confirmed the absence of the *rhaR* transcript in all the deletion strains (AR225, AR227 and AR237) and its recovery in the re-transformation (AR256) strain (Additional file 3: Figure S3). Overexposure of gels revealed the presence of trace amounts of the *rhaA* and *rhaE* transcripts in the ∆*rhaR* strains under inducing conditions, therefore RhaR does not determine these basal levels of transcription. As the artificial substrate *p*NPR was employed to evaluate α-L-rhamnosidase activity (Figure 3A) and RhaE is the principal α-L-rhamnosidase that degrades this compound [11], this low basal level of *rhaE* expression might account for the residual α-L-rhamnosidase activity observed in ∆*rhaR* strains. It should also be noted that under inducing conditions the transcript levels of *rhaE* in AR4, AR198 and AR256 did not vary and this is also in agreement with the activity data. These results show that induction of the α-L-rhamnosidase structural genes by L-rhamnose is dependent on the transcriptional activator RhaR.

### Deletion of *rhaR* affects vegetative growth on L-rhamnose and its metabolism

**Figure 4:**
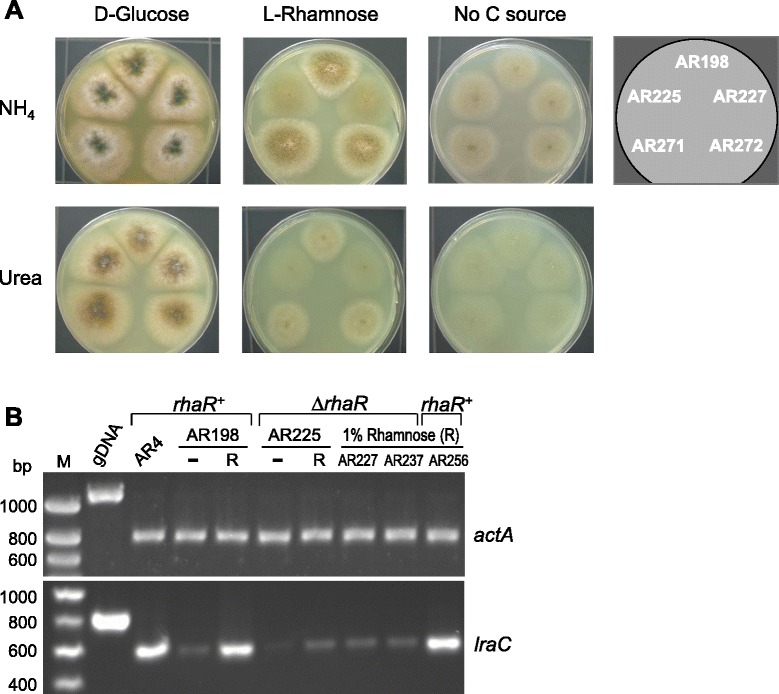
**Disruption of** ***rhaR*** **leads to reduced induction of catabolic genes and reduced growth on L-rhamnose. (A)** Vegetative growth of ∆*rhaR* mutants on different nutrient sources. Equal numbers (3 × 10^3^/3 μl) of spores of the *rhaR* ^+^ untransformed strain (AR198), the ∆*rhaR* strains (AR225 and AR227) and the *riboB2*::Af*riboB* nutritional controls (AR271 and AR272) were spotted on solid media containing different carbon and nitrogen sources and incubated for 5 days. **(B)** Expression of *lraC* under different growth conditions (−, no carbon source; R, rhamnose) and in diverse *rhaR* genetic backgrounds. Total RNA was reverse transcribed and subjected to amplifications using specific primers for *actA* (control) and *lraC*. Amplifications were reduced to 22 cycles in order to obtain semi-quantitative data.

The extent to which disruption of *rhaR* affects the ability of the fungus to grow on L-rhamnose was studied by plating the two *A. nidulans* ∆*rhaR* mutants (AR225, AR227), the untransformed strain (AR198) and the riboflavin nutritional controls AR271 and AR272 on minimal media containing either 1% w/v glucose or 1% w/v L-rhamnose as the sole carbon sources. As nitrogen sources, both urea (a poor source) and ammonium tartrate (a preferred source that can be also used as a poor carbon source) were evaluated. Whereas the ∆*rhaR* strains grew as well as the controls on glucose media this was severely reduced in the presence of L-rhamnose alone, the sparse growth observed resembling that seen on control plates lacking sugars (Figure 4A). Thus it can be concluded that the transcriptional activator RhaR not only regulates the expression of the enzymes that liberate the inducing carbon source L-rhamnose from complex substrates, but also controls the assimilation of this sugar.

As noted before (Figure 1B), *rhaR* is divergently transcribed from the adjacent *lraC* gene in filamentous ascomycetes including *A. nidulans* thus suggesting a regulatory connection between them. To ascertain whether the reduced growth of the ∆*rhaR* strains on L-rhamnose is due to RhaR-regulated expression of the L-rhamnose catabolic pathway the *lraC* gene was selected for analysis. RT-PCR showed that *lraC* has a similar pattern of expression to those of the rhamnosidase genes *rhaA* and *rhaE*: while a basal level of *lraC* mRNA is observed in strain AR198 in the absence of any carbon source and induction of its expression clearly occurs on L-rhamnose, the ∆*rhaR* strains (AR225, AR227 and AR237) failed to show increased transcript levels in the presence of the inducer (Figure 4B); as expected, L-rhamnose-dependent *lraC* transcript synthesis was observed for the retransformation strain AR256. RhaR is therefore involved in L-rhamnose catabolism by virtue of its influence on the expression of *lraC* and hence confers *A. nidulans* the ability to adapt its metabolism to the availability of L-rhamnose.

The data presented reveal that deletion of *rhaR* not only leads to reduced induction of the α-L-rhamnosidase genes by L-rhamnose but also to reduced induction of the catabolic gene *lraC*. As a result, *rhaR* loss-of-function mutants exhibit severely impaired growth on L-rhamnose. In this regard, the pathway for the degradation of L-rhamnose in *P. stipitis* was found to be induced by L-rhamnose [29], and more recently, transcriptome analysis carried out in this yeast has revealed that all the catabolic genes are up-regulated in the presence of this sugar [16]. While this work was being completed the *Aspergillus niger* L-rhamnose regulator was identified and microarray analysis also showed it to be involved in L-rhamnose release and catabolism [30].

### Expression of *rhaR* is neither repressed by glucose nor induced by L-rhamnose

**Figure 5:**
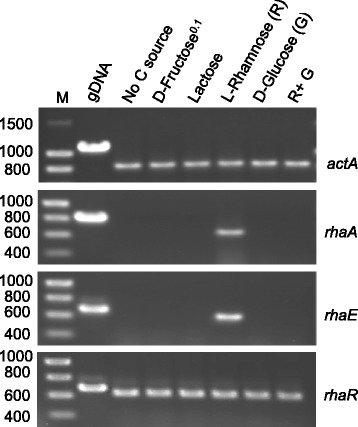
**Expression of** ***rhaA*** **,** ***rhaE*** **and** ***rhaR*** **in a wild type strain in the presence of diverse carbon sources.** Total RNA was isolated from the wild type *biA1* strain AR5 after 18 h of pregrowth in 0.1% fructose and transfer to inducing (1% L-rhamnose), inducing-repressing (1% L-rhamnose + 1% glucose; R + G), non-inducing-repressing (*i.e.* 0.1% fructose, 2% lactose and 1% glucose), and starvation (no C source) conditions. Amplifications were reduced to 23 (*actA*), 24 (*rhaA*), 30 (*rhaE*) and 32 (*rhaR*) cycles in order to obtain semi-quantitative data. The constitutively expressed *actA* gene was included as a control.

We have previously reported that the α-L-rhamnosidase genes *rhaA* and *rhaE* in *A. nidulans* are induced by L-rhamnose and repressed by glucose as a consequence of glucose blocking rhamnose uptake, and that the glucose repression is not relieved by the *creA*^*d*^*30* mutation [11] suggesting the involvement of a novel CreA-independent mechanism of carbon catabolite repression (CCR). Given that glucose repression can occur at different levels including the repression of transactivator gene expression*,* we sought to investigate the expression profile of *rhaR* under different growth conditions. To examine whether the transcription of this gene is subject to carbon source regulation (*i.e.* rhamnose induction and glucose repression), total RNA was isolated from mycelia that were initially grown on 0.1% w/v fructose and then transferred to MM supplemented with diverse single or mixed carbon sources (0.1% fructose, 2% lactose, 1% glucose, 1% rhamnose, 1% rhamnose +1% glucose), or no carbon source, and analyzed by semiquantitative RT-PCR. The expression profiles of *rhaA* and *rhaE* were included as references and were in agreement with the data obtained by northern blot analysis [11]. Figure 5 shows that in the wild type strain steady-state levels of the *rhaR* transcript accumulate to similar levels under all the conditions tested indicating that expression of *rhaR* is neither autoregulated nor repressed by glucose. Therefore, glucose repression of the α-L-rhamnosidase genes *rhaA* and *rhaE* by a novel CreA-independent mechanism does not occur via a transcriptional cascade similar to that shown for the carbon catabolite repressor CreA and the pathway specific activators AlcR or XlnR that regulate ethanol and xylose utilization in *A. nidulans*, respectively [31,32]. That the transcription of both *rhaA* and *rhaE* requires the presence of *rhaR* - presumably its gene product - (Figure 3B) and that the transcripts of these genes could only be detected under inducing conditions (*rhaR* is constitutively transcribed), indicate that L-rhamnose is absolutely required for the induction of gene expression by RhaR and thus suggests post-transcriptional control in the regulation of this regulator.

As mentioned above, *rhaR* and *lraC* are divergently transcribed in *A. nidulans* and also in different species of filamentous ascomycetes (Figure 1B). Since this divergent gene arrangement may provide a mechanism to facilitate coordinated expression of both genes, it is interesting to note that in the conserved divergent *A. nidulans* gene pair *lraC*/*rhaR* the regulatory gene *rhaR* controls the transcription of the structural gene (*lraC*) - presumably via its gene product RhaR - but it does not control its own transcription. This suggests that the specific arrangement of *cis*-acting RhaR interaction site(s) controls transcription in just one direction and/or that in the absence of rhamnose, *lraC* but not *rhaR* could be maintained in a transcriptionally repressed state by a *cis*-acting mechanism possibly depending on a specific chromatin structure or the binding of a repressor.

### RhaR is a DNA-binding protein

**Figure 6:**
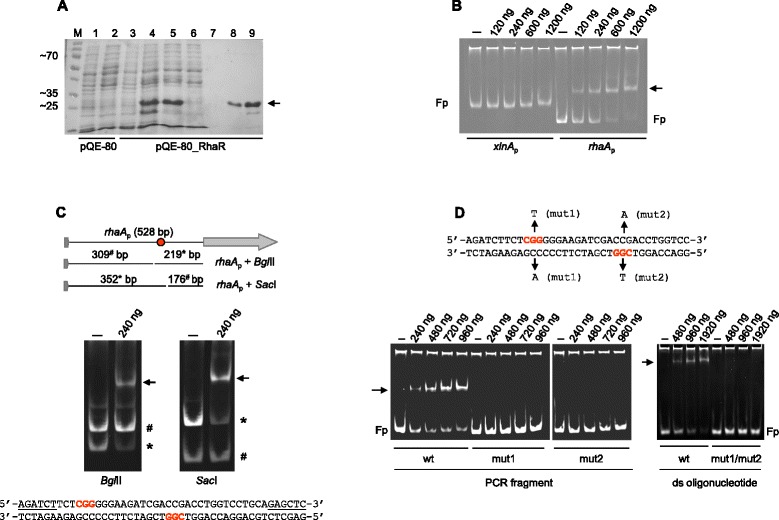
**RhaR binds to the promoter region of** ***rhaA.*** **(A)** SDS-PAGE analysis of the purification of the His_6_::RhaR (1-159*) protein expressed in *E. coli*. Lysates from uninduced and IPTG induced cells transformed with the empty plasmid (lanes 1 and 2, respectively) and the plasmid bearing the RhaR expression cassette (lanes 3 and 4, respectively). Lanes 5 and 6: Soluble fraction of the lysate before and after passage through the affinity column. Lanes 7–9: 300 mM imidazol elution fractions. The predicted molecular mass of the protein is 19,9 kDa and its position its arrowed. **(B)** Mobility shift assays of *rhaR*_p_ and *xlnA*_p_ (negative control promoter). The mobility of each PCR fragment (100 ng) was assayed in the presence of increasing amounts of His_6_::RhaR (1-159*) protein (from 0 to 1.2 μg). **(C)** Identification and delimitation of a potential binding site for RhaR in *rhaA*_p_. *rhaA*_p_ was divided into 4 overlapping fragments the sizes and locations of which are indicated in the upper part of the panel. Each restriction fragment was assayed with 0 and 240 ng of the protein. **(D)** Effect of point mutations on the proposed RhaR binding site. EMSAs using wild type and mutated fragments or double stranded oligonucleotides containing the predicted RhaR site. Fp indicates the mobility of the free probes. Arrows show the positions of the RhaR-DNA complexes.

Our results suggest that RhaR likely binds to the promoters of the three genes shown to be subject to its control. Given that under inducing conditions the *rhaA* transcript is more abundant than that of *rhaE* [11] and that compared to *lraC* the transcription of the rhamnosidase genes seems to be more dependent on RhaR (compare Figures 3B and 4B), we chose to assess RhaR binding to the *rhaA* promoter. Non-radioactive electrophoretic mobility shift assays (EMSAs) were carried out to evaluate the binding ability of an affinity-purified (Figure 6A) truncated form of RhaR (His_6_::RhaR (1-159*)) containing the entire putative DNA-binding and dimerization domains. For this purpose a PCR fragment (*rhaA*_p_) was generated that includes the 5′ region (528 bp) of the *rhaA* gene as well as the stop codon of the previous gene (AN10274). As shown in Figure 6B, *rhaA*_p_ was shifted upon incubation with increasing amounts of His_6_::RhaR (1-159*) indicating that RhaR is a DNA binding protein. Sequence specificity of the DNA-binding activity was indicated by the absence of band shift with a PCR fragment (*xlnA*_p_; [33]) corresponding to the unrelated promoter of the xylanolytic *xlnA* gene which is regulated by the Zn_2_Cys_6_ transcriptional activator XlnR [32]. To further characterise binding, overlapping fragments of the *rhaA* promoter and site-destroying mutated targets were employed (see below).

EMSA was carried out on the restriction enzyme digestion products of the 528 bp *rhaA*_p_ fragment with Bgl*II* (yields fragments of 309 and 219 bp) and Sac*I* (yields fragments of 352 and 176 bp). The 219 Bgl*II* and 352 Sac*I* bp fragments are clearly shifted, indicating that the RhaR target site within the *rhaA* promoter maps within a 45 bp fragment flanked by the Bgl*II* and Sac*I* sites (Figure 6C). Interestingly, within this region exists a single putative site of the form CGG-X_11_-CCG which is identical to the proposed Gal4 binding sites [34].

Gal4 has a stringent requirement for the central base pair of the CGG inverse repeats [35]. In order to investigate whether the site identified could also be a DNA target for RhaR, we carried out an EMSA analysis in which three 219 bp PCR-amplified promoter fragments containing one or two point mutations within the CGG triplets were compared to the wild type consensus site. As shown in Figure 6D, mutations (G to T) at the central position of each CGG triplet severely impair binding, indicating that both sites are important and possibly recognized by a RhaR dimer molecule. As expected, identical results were obtained when the *rhaA* promoter fragment combining both mutations was tested (not shown). To further confirm these results, EMSAs were performed using double-stranded 35-mer oligonucleotide probes containing the predicted RhaR site of *rhaA* flanked by 9 bp (Figure 6D). Specific binding to this site was confirmed by the absence of band shift with a doubly mutated 35-mer oligonucleotide carrying point mutations (G to T) in the central position of the two GCC triplets, compared to the shift observed for the non-mutated oligonucleotide. Taken together these results confirm RhaR to be a DNA binding protein and identify a candidate DNA sequence for RhaR recognition which is in good agreement with target sites recognized by other Zn(II)_2_Cys_6_ binding proteins.

### Conservation of RhaR among fungi

**Figure 7:**
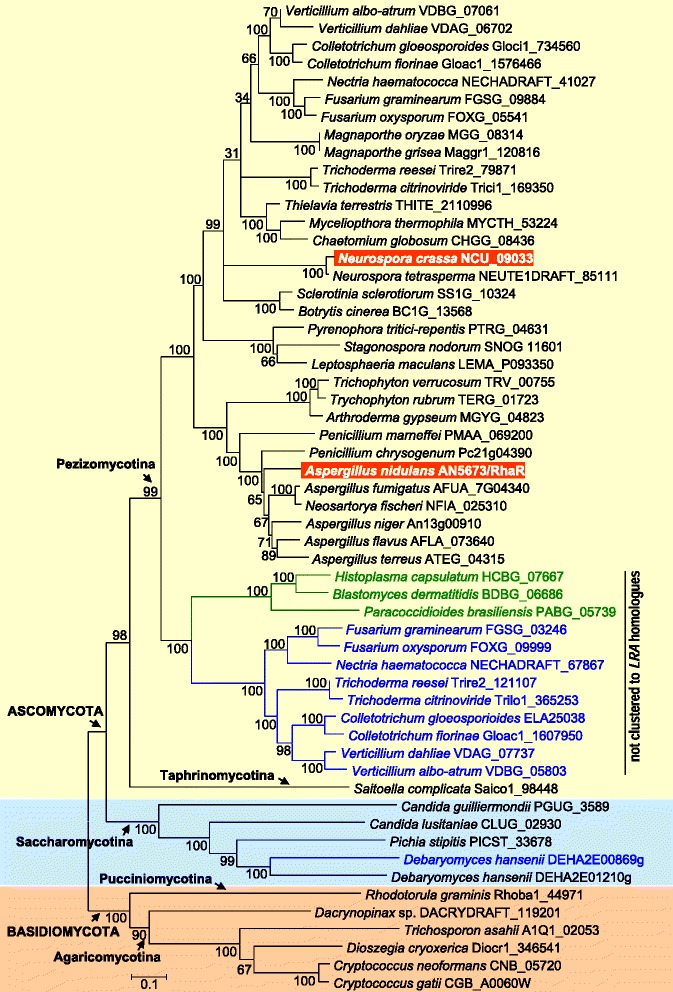
**Phylogenetic tree of selected** ***A. nidulans*** **RhaR homologues.** Homologues were identified by BLAST searches in GenBank and sequencing programs. Alignment was done using ClustalW. Tree reconstruction was generated from the alignments using the neighbour-joining method. MEGA3 software was used to carry out the analysis. Bootstrap values are adjacent to each internal node, representing the percentage of 1000 bootstrap replicates. Protein identifiers correspond to the Sequencing Centre accession numbers. Species in green letters only contain the non-clustered *rhaR* gene; species in blue contain both the clustered and non-clustered genes.

Given the identification of *rhaR* as a gene that encodes a transcriptional activator of the *A. nidulans* L-rhamnose regulon, we were prompted to search for other fungal proteins potentially involved in regulation by this carbon source. Whilst homologues to this protein can be found across a diversity of species in ascomycetes and basidiomycetes, no clear homologues were found in zygomycetes and chytridiomycetes, suggesting that RhaR emerged after the divergence of the dikarya group from the basal fungi. Phylogenetic analysis using the amino acid sequence of *A. nidulans* RhaR (Figure 7) and a number of predicted sequences downloaded from genome sequencing centres showed a broader distribution among ascomycetes than in basidiomycetes, and that RhaR orthologs can be found in the three subphyla of the ascomycetes (Saccharomycotina, Taphrinomycotina and Pezizomycotina) which include a number of important pathogens as well as industrially useful species. In ascomycetes, bootstrap values reveal the occurrence of two distinct classes of genes encoding RhaR (Figure 7), one of which is typified by that of *A. nidulans* and is the object of this work. The second class of RhaR is encoded by a single *rhaR* gene in *Histoplasma capsulatum*, *Blastomyces dermatiditis* and *Paracoccidioides blasiliensis,* whereas Fusarium, Nectria, Trichoderma, Colletotrichum and Verticillium species have both types of *rhaR* in their genomes. It is noteworthy that the most ubiquitous copy of *rhaR* in these fungi maps close to the L-rhamnose catabolic pathway genes whereas the *rhaR* counterpart is located outside the cluster, suggesting possible functional specialization of the latter. Another amplification event - increasing from one copy of *rhaR* to two tandem paralogs *-* occurred in the spoilage yeast *Debaryomyces hansenii*. In this yeast, DEHA_01210 is closer to a potential α-L-rhamnosidase gene (DEHA_1276) and to *LRA4* (DEHA_1430) than DEHA_0869. In the Taphrinomycotina taxon, we found only one homologous gene in the anamorphic saprobic yeast *Saitoella complicate*; the only other genome data available in Taphrinomycotina being that of *Schizosaccharomyces*. Among the basidiomycetes, the gene seems to be present in a few members of the subphyla Agaricomycotina and Pucciniomycotina, suggesting gene loss in some lineages.

## Conclusions

L-Rhamnose released from the degradation of plant cell wall materials induces the production of diverse enzymes (*e.g*. α-L-rhamnosidases, endopolygalacturonases, exo-rhamnogalaturonan hydrolases and rhamnogalacturonan acetylesterases) appropriate for the continued depolymerisation/modification and utilisation of these substrates. In filamentous fungi, production of these enzymes has been shown to be induced by L-rhamnose. The *A. nidulans* Zn(II)_2_Cys_6_ DNA binding transcription factor RhaR positively regulates the expression of both the enzymes that liberate L-rhamnose from complex substrates (α-L-rhamnosidases) as well as those involved in the assimilation of this sugar.

Homologues to RhaR can be found across a diversity of ascomycetes which include a number of important pathogens as well as industrially useful species. The findings presented in this work thus provide valuable information with potential for the design of strategies with which to modulate α-L-rhamnosidase production in fungi and may well be of relevance to the production of pectin degrading enzymes, a very important group of food-use enzymes some genes of which are induced by L-rhamnose and could hence share common regulatory elements. In this context, expression studies in *A. niger* have shown that the rhamnogalacturonan acetylesterase encoding gene *rgaeA* was not only expressed in the presence of D-galacturonic acid, polygalacturonate and sugar beet pectin, but also in the presence of L-rhamnose [36]. Positively acting systems responding to L-rhamnose have also been suggested for the *Aspergillus aculeatus* rhamnogalacturonan hydrolase RhgA and the *A. niger* exo-rhamnogalacturonan hydrolases RgxA, RgxB and RgxC [37,38].

The data obtained may also have relevance in phytopathology since the α-L-rhamnosidase activity of the cereal pathogen *Stagonospora avenae* has been shown to be able to detoxify plant defence saponins, antifungal molecules containing rhamnose [39]. The hydrolysis of one of the two L-rhamnose residues in potato sprout α-chaconine also seems to be the first step in the latter’s detoxification [40]. The production of endopolygalacturonase (endoPG; an enzyme that appears to play a key role in the fungus-plant interaction) by *Colletotricum lindemuthianum*, a fungal pathogen causing bean anthracnose, is also enhanced when the fungus grows in the presence of L-rhamnose as the sole carbon source, and the induction observed was greater than that obtained with pectin [41]. In *Magnapothe grisea*, genes involved in the utilization of non-preferred carbon sources, including α-L-rhamnosidase A (MGG_05246), were up-regulated during appressorium formation [42].

Finally, the data we have obtained may also have implications in human pathogenesis. A recent study carried out in the human pathogen *C. neoformans* directed towards the identification of transcriptional factors potentially involved in the formation of the capsule (a key virulence factor) identified gene CNAG_04093 (the ortholog to *rhaR* in this fungus) as a ‘first responder’ [43]. Our work could therefore eventually contribute to the characterization of targets for new antifungal compounds directed towards the inhibition of α-L-rhamnosidase activity and possibly other detoxifying activities.

## Methods

### Strains, media and growth conditions

*Escherichia coli* strain DH5α was used as the host for cloning experiments and plasmid amplification [44]. *E. coli* strain BL21 (DE3) was used for high-yield expression of the RhaR recombinant protein [44]. Fungal strains used in the study are listed in Additional file 4: Table S1. *A. nidulans* strain AR198 (relevant genotype *pyroA4*; ∆*nkuA*::*argB;* reference TN02A21) [25] was used for *rhaR* deletion. For sexual crosses and transformations with *argB*-carrying plasmids (*e.g.* pILJ16-RhaR) derived from pILJ16 [26] the *A. nidulans* AR4 strain was used*. A. nidulans* AR70 was used as a nutritional (*i.e.* arginine prototroph) control in different assays. For expression analyses, the *A. nidulans* wild type AR5 (*biA1*) strain was used. The following *N. crassa* wild type and mutant strains were used: *N. crassa* ∆NCU09033 mutant strain (FGSC 11390) was generated by the *Neurospora* Genome Project [17] and was obtained together with the wild type reference strain 74*A* (74-OR23-IV *A,* FGSC 2489) from the Fungal Genetics Stock Center at the University of Missouri-Kansas City, MO [18].

*E. coli* was grown in Luria-Bertani (LB) medium with or without 100 μg ml^−1^ ampicillin. Strains of *A. nidulans* were grown in either minimal (MM), MM + 0.5% w/v yeast extract (YMM) or complete (CM) medium [45,46] containing 1% w/v of the carbon source unless specified otherwise, and supplemented with the appropriate requirements. Except where indicated, ammonium tartrate (5 mM) was added to MM and YMM media as the nitrogen source. Strains of *N. crassa* were propagated on Vogel’s minimal medium (VM) containing 1.9% w/v sucrose or 1% L-rhamnose [47] and supplemented with 100 μg ml^−1^ histidine and 0.25 μg ml^−1^ biotin. For solid media 1.5% w/v agar was added.

For transfer experiments mycelial biomass was generated from spores (5 × 10^6^ spores ml^−1^) inoculated into YMM to which 0.1% w/v fructose was added as the sole carbon source. After ~18 h of growth at 37°C with orbital shaking at 200 rpm, mycelia were harvested, washed with MM without carbon source and transferred (1 g wet mycelium to 20 ml). Induction medium was prepared by substituting fructose with L-rhamnose at 1% w/v, and induction-repression medium by substituting fructose with rhamnose plus glucose (1% w/v each). Other carbon sources were added in equimolar amounts instead of L-rhamnose where indicated.

### α-L-Rhamnosidase assays

α-L-Rhamnosidase activity was measured using the artificial substrate *p*-nitrophenyl α-L-rhamnopyranoside (*p*NPR). The release of *p*-nitrophenol was measured spectrophotometrically at 400 nm. The assay was performed for 15 min at 50°C with shaking in a final volume of 250 μl using 1.4 mM substrate in 100 mM McIlvaine buffer (citrate-phosphate buffer) pH 4.0, essentially as described [27]. MM plates supplemented with 40 μM 4-methylumbelliferyl α-L-rhamnopyranoside (MUR) and 1 mM McIlvaine buffer pH 4.0 were used for plate assays of α-L-rhamnosidase activities. After incubation, hydrolysis of MUR was visualised under UV light.

### Transformation and nucleic acid procedures

Standard molecular techniques were as described [44]. *A. nidulans* transformations of non-homologous recombination deficient (∆*nkuA*) strains was done based on previous methods [48,49]. Protoplasts were generated using Vinoflow FCE lysing enzyme (Novozymes).

PCR reactions were performed using Phusion High-Fidelity or DyNAzyme II DNA polymerases (FINNZYMES). Restriction enzymes, Klenow enzyme, Antarctic phosphatase and T4 DNA ligase were purchased from Roche Diagnostics and New England BioLabs. All products were used as recommended by the manufacturer. Nucleic acid sequencing was carried out at the DNA Sequencing Service of the University of Valencia using the BigDye Terminator v3.1 Cycle Sequencing Kit and the ABI PRISM 310 Genetic Analyzer (Applied Biosystems). Chromatograms were analyzed using the program Chromas LITE 2.01. Oligonucleotide primers used in this study are listed in Additional file 5: Table S2. Genomic DNA from AR5 or AR198 were used as templates to generate *A. nidulans* PCR products.

Total RNA was isolated from *A. nidulans* mycelia using RNA Plus (QBiogene, USA) following the manufacturer’s instructions. RNA quantity was measured using a NanoDrop ND-1000 Spectrophotometer (Nano-Drop Technologies) and its quality assessed by agarose gel electrophoresis. RT-PCR was performed in 20 μl using 2.8 μg of total RNA, an oligo (dT)_12–18_ anchor primer, RNase OUT recombinant ribonuclease inhibitor and the MMLV SuperScript III reverse transcriptase (Invitrogen). After treating the cDNA with RNase-free DNase (Roche), amplification of cDNA targets was performed using an aliquot (4 μl; 1/5^th^) of the RT reaction and specific primers (Additional file 5: Table S2). Where possible, the sense and antisense oligonucleotides were located on either side of introns to differentiate genomic and cDNAs. The actin gene *actA* (AN6542) was used as a loading control. To confirm that PCR amplifications were in a linear range, cycle titrations were performed for each gene (not shown). From these analyses, 22–24 cycles were determined to be optimal for *actA*, *lraC* (AN5672) and *rhaA* (AN10277); 26–30 for *rhaE* (AN7151); and 28–32 for *rhaR* (AN5673).

### Generation of a *rhaR* deletion mutant and complemented strains

Deletion of *rhaR* was achieved by replacing its entire open reading frame (from −21 to +61 relative to the ATG and Stop codons respectively) with the *A. fumigatus riboB* gene (Afu1g13300) including its flanking regions (named Af*riboB*). This allele has been shown to complement the *riboB2* allele of *A. nidulans* and is not directed to any specific site in the genome of ∆*nkuA* strains [25]. A disruption cassette in which Af*riboB* was flanked on either side by the upstream and downstream untranslated regions (UTRs) of *rhaR* was generated: oligonucleotides RC6-1 and RC6-2, which include *Not*I and *EcoR*I restriction sites respectively, were used to amplify the 1755 bp 5′-UTR region; a 1463 bp 3′-UTR region of *rhaR* was amplified using oligonucleotide pair RC6-3 and RC6-4, which include the *EcoR*I and *Xho*I restriction sites respectively. Both fragments were subcloned into the *Not*I and *Xho*I sites of pBluescript SK(+) (Stratagene) to yield pBS_UTR/RhaR, and a 1.9 kb *EcoR*I fragment comprising the Af*riboB* expression cassette (obtained from plasmid p1548; [49]) was then subcloned into the same site of pBS_UTR/RhaR to generate pBSdeltaRhaR. This plasmid was subsequently digested with *Not*I and *Xho*I to release the 5.2 kb deletion cassette which was used to transform strain AR198. Riboflavin prototrophs were selected and purified, tested for α-L-rhamnosidase activity on MUR plates (Additional file 2: Figure S2A) and analysed by PCR. Additional file 2: Figure S2B shows that using the oligonucleotide pairs R0-B1 and B2-R5, PCR fragments of 2.61 kb and 1.83 kb were obtained corresponding to the expected upstream and downstream junctions in both transformants whereas a 2.25 kb fragment was observed using R0-R1 in the untransformed strain AR198. Sequencing and Southern blot analysis further confirmed the occurrence of single copy integrations at the homologous locus as well as the absence of ectopic integrations in two selected transformants (T4/AR225 and T11/AR227). In the Southern analysis, genomic DNA from the transformed (T4 and T11) and untransformed (AR198) strains was digested with *Sph*I. After probing the blot with a digoxigenin (DIG)-labelled 5′ UTR *rhaR::*Af*riboB* chimeric probe, the Δ*rhaR* strains displayed a 6.65 kb band corresponding to insertion of the 5.02 kb disruption fragment into *rhaR* whereas the parental strain yielded a 7.52 kb band (data not shown).

The Δ*rhaR* mutant AR225 (T4) was subsequently crossed to *A. nidulans* AR4 to obtain *nkuA*^*+*^, Δ*rhaR::*Af*riboB* strains of interest (H8/AR234 and H23/AR237). Diagnostic PCRs (Additional file 2: Figure S2B) and Southern analysis (not shown) confirmed single-copy integration of the Af*riboB* disruption cassette at the *rhaR* locus in both strains. The presence of the *nkuA* wild type allele (AN7753) was also confirmed via PCR using a pair of primers annealing within the ORF (not shown). Strain AR237 was chosen for further studies.

For complementation of the *rhaR* deletion mutants, the wild type *rhaR* gene, comprising the coding sequence along with its native promoter and terminator (739 and 414 bp respectively), was generated by PCR from gDNA of strain AR198 using the oligonucleotide pair RC6-7/RC6-8, and cloned into the *EcoR*V site of pILJ16 [26]. The resulting plasmid (pILJ16_RhaR) was used to transform the *nkuA*, *argB2,* Δ*rhaR::AfriboB* strain AR237. Transformant C2/AR256 was selected for growth in the absence of arginine and plasmid integration was confirmed by PCR (Additional file 2: Figure S2B) and Southern blotting (not shown).

### Construction of a riboflavin nutritional control strain

Sequencing of the *A. nidulans riboB2* allele revealed the deletion of a single cytosine residue compared to the wild type (CTT.**C**GT.CAA… to CTT.gtc.aag…) that would cause a frameshift after amino acid L285 and protein truncation after the addition of 145 novel amino acids. To construct a nutritional control strain (*i.e.* riboflavin prototroph), a *Sph*I DNA fragment (3.9 kb) containing Af*riboB* (form plasmid pTN2; [25]) flanked by the *A. nidulans riboB* (AN0670) UTR flanking regions (952 bp upstream and 926 bp downstream) was used to transform AR198 and thereby obtain an *rhaR* nutritional control strain (AR271) in which riboflavin prototrophy is conferred by Af*riboB* inserted at the *riboB* locus. The construction was confirmed by both diagnostic PCR (Additional file 6: Figure S4) and Southern analysis (not shown).

### Production of RhaR protein for *in vitro* DNA binding assays

Vectors for expressing the truncated His_6_-tagged RhaR protein (residues M1-A159*), encompassing the Zn(II)_2_Cys_6_ region as well as the predicted linker and dimerization domain, were constructed by subcloning the 476 bp *Sma*I-*Pst*I fragment obtained from pGEX-RhaR (bearing a 480 bp cDNA fragment amplified by PCR from strain AR198 using the oligonucleotide pair GST:RhaR_Sma and GST:RhaR_RI) into pQE-80 L (Qiagen) previously digested with the same enzymes to achieve the correct reading frame. The His_6_::RhaR (1-159*) protein was expressed from the T5 promoter in *E. coli* BL21 (DE3) and purified on nickel-nitrilotriacetic (NiNTA) agarose (Qiagen) using imidazole according to the recommendations from the supplier. The fraction eluted at 300 mM was directly used for electrophoretic mobility shift assays (EMSAs).

### EMSAs

The set of overlapping DNA fragments used for non-radioactive gel mobility shift experiments were amplified by PCR from the 5′ flanking region of the *rhaA* gene using various oligonucleotide primer pairs. The 528 bp wild type probe immediately upstream of the ATG codon (*rhaA*_p_) used in Figure 6B was generated using oligonucleotides ^WT^rhaA-dir1 and ^WT^rhaA-rev1. The 219 bp wild type, mut1 and mut2 promoter fragments used as a probes in the experiment shown in Figure 6D were generated using the oligonucleotide pairs ^WT^rhaA R1-dir/^WT^rhaA-rev1, ^mut1^rhaA R1-dir/^WT^rhaA-rev1 and ^mut2^rhaA R1-dir/^WT^rhaA-rev1, respectively. Other probes (see Figure 6C) were obtained from restriction digestion of *rhaA*_p_. Synthetic double-stranded wild type and double mutant oligonucleotides were generated as described previously [50] using the primer pairs ^WT^rhaA R1-dir/^WT^rhaA R1-rev and ^mut1mut2^rhaA R1-dir/^mut1mut2^rhaA R1-rev. All probes (100 ng) were incubated with increasing amounts of the His_6_::RhaR (1-159*) protein in 20 μl reaction mixtures (25 mM HEPES (pH 7.9), 50 mM KCl, 5 mM MgCl_2_, 1 mM DTT, 0.1 mM EDTA and 1 μg of salmon sperm DNA). After incubation at 4°C for 30 min DNA-protein complexes were separated on 5-8% non-denaturing polyacrylamide gels in 0.5 × TBE at 4°C (120 volts for 2–4 hours). Visualization of bands was done by ethidium bromide staining as reported [51].

### Bioinformatic analyses

Genomic data for *A. nidulans* and other fungi were provided by the Broad Institute (http://www.broad.mit.edu/annotation/fgi/), the Join Genome Institute (http://genome.jgi-psf.org/programs/fungi/index.jsf), and the National Center for Biotechnology Information (NCBI; http://www.ncbi.nlm.nih.gov/sutils/genom_table.cgi?organism=fungi). Genes were identified in these databases using BLASTp and tBLASTn searches with the predicted amino acid sequences of the *A. nidulans rhaR* or yeast *LRA* gene products. Other bioinformatic tools and software packages were provided by the ExPASy Proteomics Server (http://www.expasy.org/), the EMBOSS software suite (http://emboss.bioinformatics.nl/) and NCBI.

### Nucleotide accession number

The sequence of the *rhaR* cDNA has been deposited in the GenBank database with the accession number HG326214.

## Additional files

Additional file 1: Figure S1
**Nucleotide and amino acid sequences of the** ***A. nidulans rhaR*** **/AN5673 gene.** **(A)** Sequence of the predicted coding region of *rhaR.* Numbers refer to amino acids. Introns are indicated by red letters. The Zn(II)_2_Cys_6_ region appears highlighted in yellow and the six canonical cysteine residues are circled, the coiled-coil region in green, and the MHR in red. **(B)** Multiple sequence alignment of the *A. nidulans* XlnR Zn(II)_2_Cys_6_ binuclear cluster DNA binding domain and the same region of 12 selected XlnR homologues shown in Figure 7. Gal4 and Ppr1 are also included because of a potential similar model of bipartite DNA recognition. The alignment was performed with ClustalW2 and shading was performed with Box-shade. The residues shown in red correspond to the six conserved cysteines. Residues shown in blue correspond to the critical amino acids that in Gal4 (K17 and K18) and Ppr1 (K40 and K41) make specific contacts with bases in the highly conserved CGG triplets [13,23]. Shown in pink is the very conserved proline. Identical residues are depicted on a black background whereas similar residues are in grey background. Fully conserved, strongly similar and weakly similar residues are marked with *, : and . respectively. **(C)** Domain architecture of RhaR. Green, Zn(II)_2_Cys_6_ motif; red, fungal specific transcription factor domain. Additional file 2: Figure S2
**Identification and gene replacement analyses of the** ***A. nidulans*** **∆** ***rhaR*** **mutants.** **(A)** Detection of α-L-rhamnosidase activity in untransformed (AR198), riboflavin nutritional control (AR271) and selected mutant (T4, T6, T7, T10, T11 and T12) strains. **(B)** The correct replacement of AN5673/*rhaR* with the Af*riboB* expression cassette in the mutants was verified by the absence or appearance of PCR products of the expected size using gene specific primers (R1-R3, B1 and B2) and two primers (R0 and R5) located outside of the flanking sequences of *rhaR* used in the gene replacement cassette. An schematic diagram of the *rhaR* locus and gene replacement events are shown. **(C)** α-L-Rhamnosidase plate assay of selected ∆*rhaR* progeny (H8, H23, H12 and H19) from the cross AR225 × AR4. **(D)** Detection of α-L-rhamnosidase activity in *rhaR* complemented (C2, C33, C35) and control (AR4, T4 and H23) strains. Additional file 3: Figure S3
***rhaR*** **expression in** ***rhaR*** ^**+**^ **and** **∆** ***rhaR*** **strains.** RT-PCR analyses for *rhaR* in selected *rhaR* ^+^ (AR4, AR198 and AR256) and ∆*rhaR* (AR225, AR227 and AR237) strains under the same conditions used in Figure 3. Additional file 4: Table S1. List of *Neurospora crassa* and *Aspergillus nidulans* strains used in this study. Additional file 5: Table S2. List of primers used in this study. Additional file 6: Figure S4
**Construction and gene replacement analysis of the riboflavin nutritional control strains** ***riboB2*** **::Af** ***riboB.*** **(A)** The correct replacement of AN0670/*riboB* with the Af*riboB* expression cassette in the nutritional control complemented strains (AR271-AR273, AR279) was verified using gene specific primers (N1, N2, F1-F4) and primers located outside of the gene replacement cassette (N0, N3). **(B)** Schematic diagram of the *riboB* locus and gene replacement events are shown.

## Abbreviations

CAZy: Carbohydrate-active enzymes
CCR: Carbon catabolite repression
CM: Complete medium
EMSA: Electrophoretic mobility shift assay
GH: Glycosyl hydrolase
MM: Minimal medium
MUR: 4-methylumbelliferyl α-L-rhamnopyranoside
ORF: Open reading frame
PCR: Polymerase chain reaction
*p*NPR: *p*-nitrophenyl α-L-rhamnopyranoside
TF: Transcription factor
UTR: Untranslated region
