# Supplementary material for: The Aspergillus nidulans Zn(II)2Cys6 transcription factor AN5673/RhaR mediates L-rhamnose utilization and the production of α-L-rhamnosidases
Source: Microb Cell Fact. 2014 Nov 22;13:161. doi: 10.1186/s12934-014-0161-9 (PMC4245848; doi:10.1186/s12934-014-0161-9)

A

```
atgccgaacgctgctatcgcagctgtaccgcaacaaccccgccttcagcttcaaattcg
 M  P  N  A  A  I  A  A  V  P  Q  Q  P  P  P  S  A  S  N  S     20
gcatttgaccaggacaaagaccacagtgctgatgagtcctggtcggctgtcgatagtgat
 A  F  D  Q  D  K  D  H  S  A  D  E  S  W  S  A  V  D  S  D     40
gccggtcctgctcaaaatggatcctcgcgtggcttgaaacgcaggcggccactcacagtc
 A  G  P  A  Q  N  G  S  S  R  G  L  K  R  R  R  P  L  T  V     60
tcgtaagtcaaccgcccgtccccggcatatccgaccagttcttcctgccctacagtgtct
 S                                                               61
tgcagtgcttgcaatgccttgcaaccttcttgcataaccatagacctaccgcgtccctct

cggttccctctttagacgctagattgacaagtctaggtgtgaattatgcaagcagcgcaa
                                     C  E  L  C  K  Q  R  K     69
ggttaaatgcggtatgttctgtggtatcctgtccttgtcctggtatctgactggcgtcta
   V  K  C                                                      72
gatcgagctcaaccaagctgtggatggtgtacacggaatggtcaggtgtgtgagtacaag
 D  R  A  Q  P  S  C  G  W  C  T  R  N  G  Q  V  C  E  Y  K     92
gaaagaaagaagcccggccttcgtgcagggtacggaaaagagctagagcaacgcctaggt
 E  R  K  K  P  G  L  R  A  G  Y  G  K  E  L  E  Q  R  L       111
acgggattcccggttacgacctatgcaccttctcattgacatttaatagacagactggag
                                                D  R  L  E     115
gatatcatccaggcgcaagctcgtctcattgagacgcatatccttcaagggcagccgcgg
 D  I  I  Q  A  Q  A  R  L  I  E  T  H  I  L  Q  G  Q  P  R    135
ctctcgatgtcgaatgatctacaccagcaaggacccctatcgtatagctcaccctcagag
 L  S  M  S  N  D  L  H  Q  Q  G  P  L  S  Y  S  S  P  S  E    155
ccttctgcagcacatgggcccagtccgcgaaatccgttctatttccatgagccgtcatct
 P  S  A  A  H  G  P  S  P  R  N  P  F  Y  F  H  E  P  S  S    175
gtgccctcagctccacgtccgccggagcctgtcactactagtccaacggacatgtcggta
 V  P  S  A  P  R  P  P  E  P  V  T  T  S  P  T  D  M  S  V    195
aaaaactctatccagaaccaaatcaccggtgctctcccctcagtaacgcccttgccacat
 K  N  S  I  Q  N  Q  I  T  G  A  L  P  S  V  T  P  L  P  H    215
ataacgaacaccacgccgcagaacgactacaacgataacgagtcatccttaaaagtacca
 I  T  N  T  T  P  Q  N  D  Y  N  D  N  E  S  S  L  K  V  P    235
gtcaatttgtttcgaaccaagaacagtcgttctctgaccccgagctcgatcttcccct
 V  N  L  F  S  N  Q  E  Q  S  F  S  D  P  E  L  D  L  P  P    255
tatgatttactgtatgcgctggttgacctctactttgagcacctaaatacttggtgcccg
 Y  D  L  L  Y  A  L  V  D  L  Y  F  E  H  L  N  T  W  C  P    275
atcctgcatcgacgaacgacacttgacactttcttcggtcccactcctttagaggaagct
 I  L  H  R  R  T  T  L  D  T  F  F  G  P  T  P  L  E  E  A    295
gaccgcatggttctctacgctatcgtcgcgactactcttcgcttttctaatgacagccga
 D  R  M  V  L  Y  A  I  V  A  T  T  L  R  F  S  N  D  S  R    315
ctcaccgaacaaaatcgcaagcggtatcacgactcctccaagcaaaaggttctgctttac
 L  T  E  Q  N  R  K  R  Y  H  D  S  S  K  Q  K  V  L  L  Y    335
ggcctggaaaactcgtccgtaaaggcactgcaggctctggtgattctcgccctggacctt
 G  L  E  N  S  S  V  K  A  L  Q  A  L  V  I  L  A  L  D  L    355
gttgggtcatctaacgggccccctggttggaaactccttgccttgataacaaggtcagtg
 V  G  S  S  N  G  P  P  G  W  K  L  L  A  L  I  T  R  S  V    375
gttcaattgggtctggccgtcgagtccaagtcaacccttataactcccctctatccttca
 V  Q  L  G  L  A  V  E  S  K  S  T  L  I  T  P  L  Y  P  S    395
atatatacattgcgggccgtcactctccccgaagcggagtcgtggattgaagacgagggc
 I  Y  T  L  R  A  V  T  L  P  E  A  E  S  W  I  E  D  E  G    415
aggcgtcggcttttctggatggtgtaccttcttgaccggtattctactctcgcaaccgct
 R  R  R  L  F  W  M  V  Y  L  L  D  R  Y  S  T  L  A  T  A    435
tttgactttgctttggatgacaaagacatcgaccgcaagcttccgtgtaaagacgagttt
 F  D  F  A  L  D  D  K  D  I  D  R  K  L  P  C  K  D  E  F    455
ttcattaagaaccagccagttgagactaggtggtttcagtcgtcgaatgaccgcccagat
 F  I  K  N  Q  P  V  E  T  R  W  F  Q  S  S  N  D  R  P  D    475
```

```
catctcattcgttccgagaacgttggttcattcggactctacgtggaaattcttggtatc
 H  L  I  R  S  E  N  V  G  S  F  G  L  Y  V  E  I  L  G  I   495
ctttcacggatacatgtattcctcaagcggcccgttgacattggggttctatctgatgtc
 L  S  R  I  H  V  F  L  K  R  P  V  D  I  G  V  L  S  D  V   515
gaggagtggcaagccacgtatcgcaaattggatagcgagttgactacctgggagttcaat
 E  E  W  Q  A  T  Y  R  K  L  D  S  E  L  T  T  W  E  F  N   535
cttcctacagagtacacctacgaaaacgcctctcgggcgtttagcggctcgaagcacaag
 L  P  T  E  Y  T  Y  E  N  A  S  R  A  F  S  G  S  K  H  K   555
gggcaccactgtgattgggttcagttacattctgtttaccagacgtgagtcaatgtttcc
 G  H  H  C  D  W  V  Q  L  H  S  V  Y  Q  T                  570
taaaacgttttcgctgttcgctgaccccaactgcagggcggtaattcggcttcattcttc
                                    A  V  I  R  L  H  S  S   578
agcagcatatcccactacacggtctccgatcttcacgccgtcgtacagtgcgagccagcg
  A  A  Y  P  T  T  R  S  P  I  F  T  P  S  Y  S  A  S  Q  R 598
atgtctgctcgcagtcgataacattgtttcagtcacccgttttgtcgtgaacaataacat
  C  L  L  A  V  D  N  I  V  S  V  T  R  F  V  V  N  N  N  I 618
tttggacaagcttggaccgccgtttgcatttacactttgggtttctgctcggttgctgct
  L  D  K  L  G  P  P  F  A  F  T  L  W  V  S  A  R  L  L  L 638
cgtccacggatctacaatcgcgcacacagtcagtcccgatatcgtgtttatcgttgatac
  V  H  G  S  T  I  A  H  T  V  S  P  D  I  V  F  I  V  D  T 658
tctttctcaaatgggcaagtactggaaagtagcggagaggtacagctcaatcctgcaacg
  L  S  Q  M  G  K  Y  W  K  V  A  E  R  Y  S  S  I  L  Q  R 678
agttctcgatgaatacggcgagtatcagcaatctggtgccggcgatggtgatcggtccac
  V  L  D  E  Y  G  E  Y  Q  Q  S  G  A  G  D  G  D  R  S  T 698
accttcttctgtcaaaatccttgcagacatgcgccgatgcgcgtttgacctggacttcct
  P  S  S  V  K  I  L  A  D  M  R  R  C  A  F  D  L  D  F  L 718
gatatcgcggcaaccccgttcgtcgccaaccgcaagtcaggcagcggctccgtcagctgg
  I  S  R  Q  P  R  S  S  P  T  A  S  Q  A  A  A  P  S  A  G 738
attgccatcgagaagcttagcacctaatgagttggagtatctagacgttttcggtttctt
  L  P  S  R  S  L  A  P  N  E  L  E  Y  L  D  V  F  G  F  F 758
caatgtcccgcgagtccccgccggtcggacccctgatattagcggtcttgacatggccga
  N  V  P  R  V  P  A  G  R  T  P  D  I  S  G  L  D  M  A  E 778
agctgtcaataaccccatgtcgatacctggattgactggaactggcagttcgaacccttt
  A  V  N  N  P  M  S  I  P  G  L  T  G  T  G  S  S  N  P  L 798
gccggtggataatgcttctacccatacaaacgagttcaatatcacgaattatttgatacc
  P  V  D  N  A  S  T  H  T  N  E  F  N  I  T  N  Y  L  I  P 818
cacccccgaaacggactggctgtttcgccccgggggttag
  T  P  E  T  D  W  L  F  R  P  G  G  *                      830
```

B

```
Myceliopthora thermophila MYCTH_53224           SVSCELCKQRKVKCDRGQPSCGWCSRNGALCEYK  97
Colletotrichum gloeosporoides Gloci1_734560     SVSCELCKQRKVKCDRGQPSCGWCSRNGALCEYK 112
Magnaporthe oryzae MGG_08314                    SVSCELCKQRKVKCDRGQPSCGWCSRNGASCEYK  94
Neurospora crassa NCU_09033/RhaR                SVSCETCKTRKVKCDRGQPACGWCSRNTVVCEYK 114
Trychophyton rubrum TERG_01723                  MVSCELCKQRKVKCDRSQPSCGWCARNGHPCEYK 106
Aspergillus nidulans AN5673/RhaR                TVSCELCKQRKVKCDRAQPSCGWCTRNGQVCEYK  92
Blastomyces dermatitidis BDBG_06686             TTSCELCRTRKVKCDRAEPSCGWCTRNGRVCIYR  78
Colletotrichum gloeosporioides ELA25038         KTSCELCKARKVKCDRAEPACSWCARHNRTCVYL  73
Saitoella complicata Saico1_98448               LTACDSCKARKVKCDRAQPSCEWCRKVGVSCVYQ  65
Candida lusitaniae CLUG_02930                   SKACNSCKEKKKKCDRASPRCGYCARNGLTCKYR  43
Debaryomyces hansenii DEHA2E00869g              SRSCDYCKLKKVKCDQTKPNCNYCIKHNESCVYS  62
Debaryomyces hansenii DEHA2E01210g              SRSCDFCKSKKVKCDGNKPSCNYCLNHGENCVYS  61
Criptococcus neoformans CNB_05720               SITCAPCRGKKIKCDSTKPTCLNCAKSPDSCYYP  60
Saccharomyces cerevisiae Gal4                   EQACDICRLKKLKCSKEKPKCAKCLKNNWECRYS  41
Saccharomyces cerevisiae Ppr1                   RTACKRCRLKKIKCDQEFPSCKRCAKLEVPCVSL  64
                                                  :*  *: :* **.   * *  *  .   *
```

Base-pair contacting residues in Gal4 & Ppr1

C

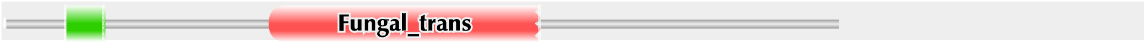

Supplement: Additional file 1: Figure S1 — Nucleotide and amino acid sequences of the A. nidulans rhaR /AN5673 gene. (A) Sequence of the predicted coding region of rhaR. Numbers refer to amino acids. Introns are indicated by red letters. The Zn(II)2Cys6 region appears highlighted in yellow and the six canonical cysteine residues are circled, the coiled-coil region in green, and the MHR in red. (B) Multiple sequence alignment of the A. nidulans XlnR Zn(II)2Cys6 binuclear cluster DNA binding domain and the same region of 12 selected XlnR homologues shown in Figure 7. Gal4 and Ppr1 are also included because of a potential similar model of bipartite DNA recognition. The alignment was performed with ClustalW2 and shading was performed with Box-shade. The residues shown in red correspond to the six conserved cysteines. Residues shown in blue correspond to the critical amino acids that in Gal4 (K17 and K18) and Ppr1 (K40 and K41) make specific contacts with bases in the highly conserved CGG triplets [13,23]. Shown in pink is the very conserved proline. Identical residues are depicted on a black background whereas similar residues are in grey background. Fully conserved, strongly similar and weakly similar residues are marked with *, : and . respectively. (C) Domain architecture of RhaR. Green, Zn(II)2Cys6 motif; red, fungal specific transcription factor domain. [file 12934_2014_161_MOESM1_ESM.pdf]
