# Supplementary material for: The Aspergillus nidulans Zn(II)2Cys6 transcription factor AN5673/RhaR mediates L-rhamnose utilization and the production of α-L-rhamnosidases
Source: Microb Cell Fact. 2014 Nov 22;13:161. doi: 10.1186/s12934-014-0161-9 (PMC4245848; doi:10.1186/s12934-014-0161-9)
Supplement: Additional file 2: Figure S2 — Identification and gene replacement analyses of the A. nidulans ∆ rhaR mutants. (A) Detection of α-L-rhamnosidase activity in untransformed (AR198), riboflavin nutritional control (AR271) and selected mutant (T4, T6, T7, T10, T11 and T12) strains. (B) The correct replacement of AN5673/rhaR with the AfriboB expression cassette in the mutants was verified by the absence or appearance of PCR products of the expected size using gene specific primers (R1-R3, B1 and B2) and two primers (R0 and R5) located outside of the flanking sequences of rhaR used in the gene replacement cassette. An schematic diagram of the rhaR locus and gene replacement events are shown. (C) α-L-Rhamnosidase plate assay of selected ∆rhaR progeny (H8, H23, H12 and H19) from the cross AR225 × AR4. (D) Detection of α-L-rhamnosidase activity in rhaR complemented (C2, C33, C35) and control (AR4, T4 and H23) strains. [file 12934_2014_161_MOESM2_ESM.ppt]

## Slide 1
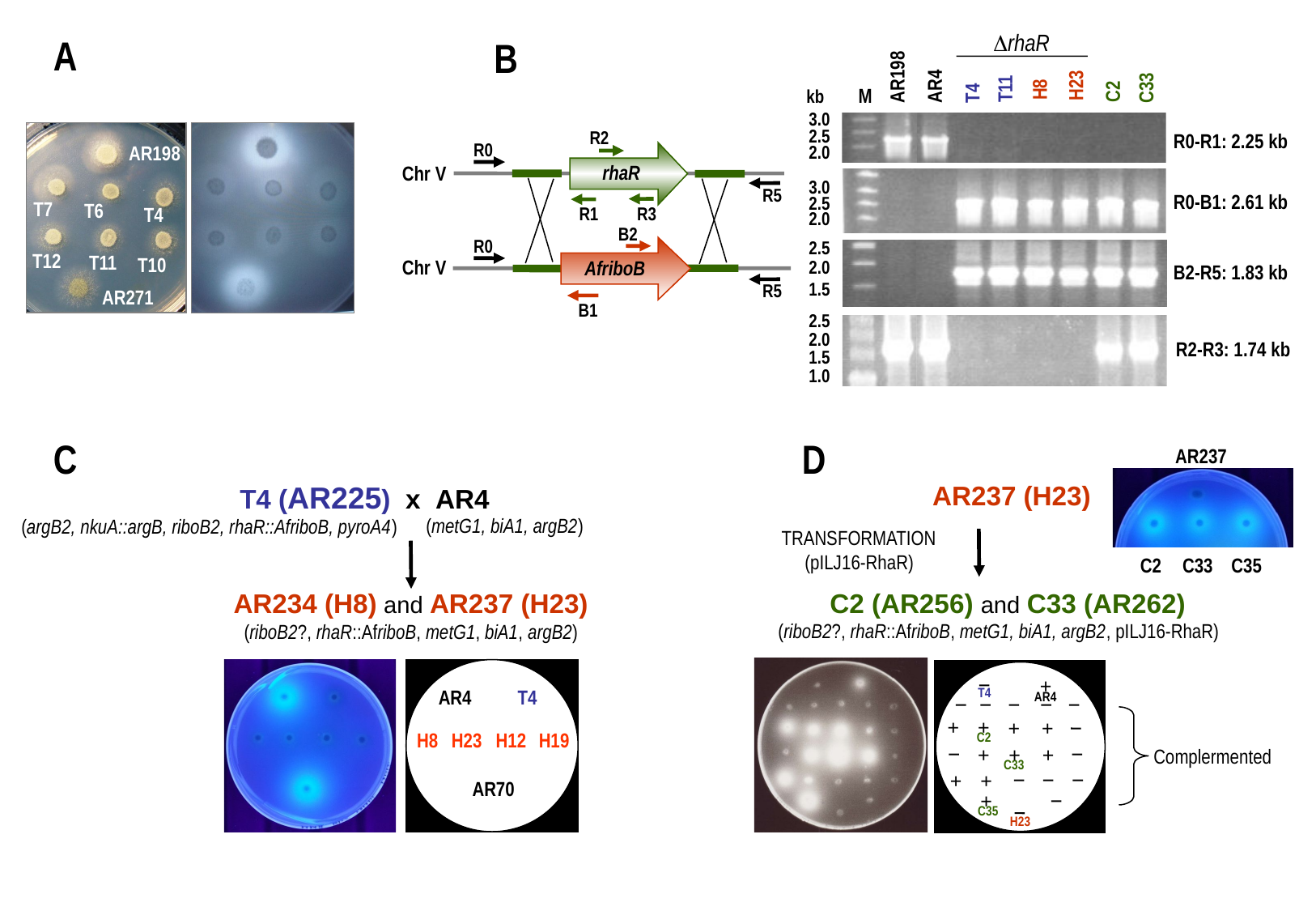

ΔrhaR
A
B
AR198
H23
AR4
C33
T11
H8
C2
T4
M
kb
3.0
2.5
R2
R0
R5
rhaR
Chr V
R1
R3
B2
R0
R5
Chr V
AfriboB
B1
R0-R1: 2.25 kb
AR198
T7
T6
T4
T12
T11
T10
AR271
2.0
3.0
R0-B1: 2.61 kb
2.5
2.0
2.5
2.0
B2-R5: 1.83 kb
1.5
2.5
2.0
R2-R3: 1.74 kb
1.5
1.0
D
C
AR237
T4 (AR225) x AR4
AR237 (H23)
 (metG1, biA1, argB2)
(argB2, nkuA::argB, riboB2, rhaR::AfriboB, pyroA4)
TRANSFORMATION
 (pILJ16-RhaR)
C2
C33
C35
 C2 (AR256) and C33 (AR262)
 (riboB2?, rhaR::AfriboB, metG1, biA1, argB2, pILJ16-RhaR)
AR234 (H8) and AR237 (H23)
(riboB2?, rhaR::AfriboB, metG1, biA1, argB2)
AR4
T4
H8
H23
H12
H19
AR70
+
T4
AR4
+
+
+
+
C2
+
+
+
Complermented
C33
+
+
+
C35
H23
