# Supplementary figures and images for: The Aspergillus nidulans Zn(II)2Cys6 transcription factor AN5673/RhaR mediates L-rhamnose utilization and the production of α-L-rhamnosidases
Source: Microb Cell Fact. 2014 Nov 22;13:161. doi: 10.1186/s12934-014-0161-9 (PMC4245848; doi:10.1186/s12934-014-0161-9)

## Slide 1
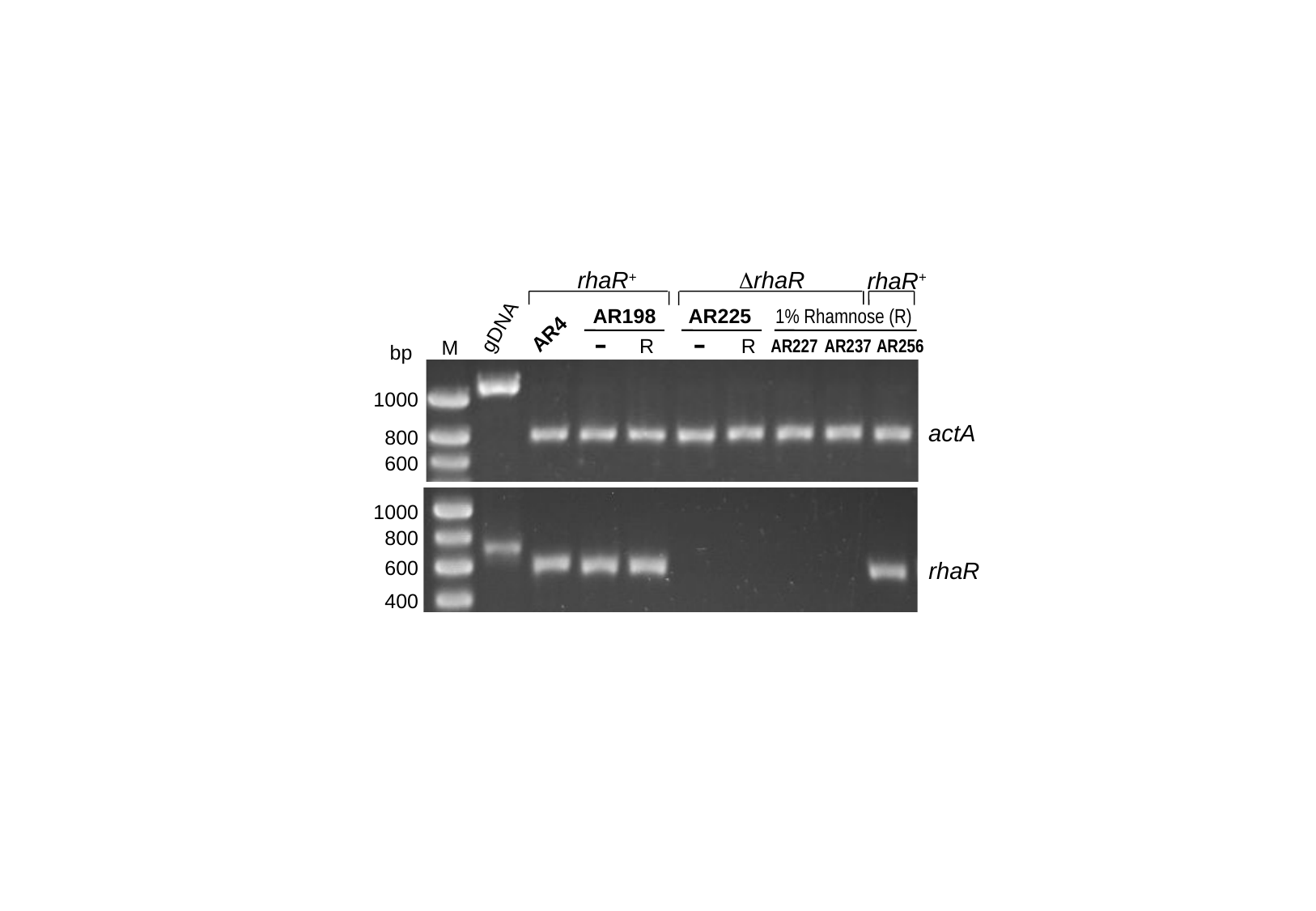

ΔrhaR
rhaR+
rhaR+
1% Rhamnose (R)
AR198
AR225
gDNA
-
-
AR4
R
R
AR227
AR237
AR256
M
bp
1000
actA
800
600
1000
800
600
rhaR
400

Supplement: Additional file 3: Figure S3 — rhaR expression in rhaR + and ∆ rhaR strains. RT-PCR analyses for rhaR in selected rhaR + (AR4, AR198 and AR256) and ∆rhaR (AR225, AR227 and AR237) strains under the same conditions used in Figure 3. [file 12934_2014_161_MOESM3_ESM.ppt]
