# Supplementary material for: The Aspergillus nidulans Zn(II)2Cys6 transcription factor AN5673/RhaR mediates L-rhamnose utilization and the production of α-L-rhamnosidases
Source: Microb Cell Fact. 2014 Nov 22;13:161. doi: 10.1186/s12934-014-0161-9 (PMC4245848; doi:10.1186/s12934-014-0161-9)
Supplement: Additional file 4: Table S1. — List of Neurospora crassa and Aspergillus nidulans strains used in this study. [file 12934_2014_161_MOESM4_ESM.pdf]

Table S1 List of *Neurospora crassa* and *Aspergillus nidulans* strains used in this study

Strain	Genotype[1]	Source
74-OR23-1V *A*	WT	FGSC#2489
AR289	ΔNCU9033	FGSC#11390
AR4	*biA1; metG1; argB2*	M. A. Peñalva (CIB/CSIC)
AR5	*biA1*	M. A. Peñalva
AR70	*biA1; metG1; argB2*::pILJ16	[7]
AR198	*pyroA4; argB2; ΔnkuA*:: *argB; riboB2*	TN02A21 [16]
AR225/T4	*pyroA4; argB2; ΔnkuA*:: *argB; riboB2; ΔrhaR*::Af*riboB*	This work
AR227/T11	*pyroA4; argB2; ΔnkuA*:: *argB; riboB2; ΔrhaR*::Af*riboB*	This work
AR234/H8	*biA1; metG1; argB2; ΔrhaR*::Af*riboB*	This work
AR237/H23	*biA1; metG1; argB2; ΔrhaR*::Af*riboB*	This work
AR256/C2	*biA1; metG1; argB2; ΔrhaR*::Af*riboB* + pILJ16-RhaR	This work
AR262/C33	*biA1; metG1; argB2; ΔrhaR*::Af*riboB* + pILJ16-RhaR	This work
AR271	*pyroA4; argB2; ΔnkuA*:: *argB; riboB2*::Af*riboB*	This work
AR272	*pyroA4; argB2; ΔnkuA*:: *argB; riboB2*::Af*riboB*	This work
AR273	*pyroA4; argB2; ΔnkuA*:: *argB; riboB2*::Af*riboB*	This work
AR279	*pyroA4; argB2; ΔnkuA*:: *argB; riboB2*::Af*riboB*	This work

[1]All the *A. nidulans* strains carry the mutant allele *veA1*
