# Supplementary material for: The Aspergillus nidulans Zn(II)2Cys6 transcription factor AN5673/RhaR mediates L-rhamnose utilization and the production of α-L-rhamnosidases
Source: Microb Cell Fact. 2014 Nov 22;13:161. doi: 10.1186/s12934-014-0161-9 (PMC4245848; doi:10.1186/s12934-014-0161-9)
Supplement: Additional file 5: Table S2. — List of primers used in this study. [file 12934_2014_161_MOESM5_ESM.pdf]

Table S2 List of primers used in this study

Name	Gene	Sequence (5'-3')[1]	Primer use
RC6-1	*rhaR*/AN5673	aaaataagaatgcggccgcGAGGAGTTCAGTGAGTCCACC	5'UTR amplification, forward
RC6-2	*rhaR*/AN5673	ggggaatTCCAACGAACAAGCTCCGTCC	5'UTR amplification, reverse
RC6-3	*rhaR*/AN5673	aagggaattcACGCCCTTTGAACGATGGAATG	3'UTR amplification, forward
RC6-4	*rhaR*/AN5673	aaccgctcgaGTGCTTGTCTTTGACTTGTCC	3'UTR amplification, reverse
pBS dir	pBS_UTR/RhaR	GTAAAACGACGGCCAGT	Sequencing of UTRs *rhaR*
pBS rev	pBS_UTR/RhaR	CAGGAAACAGCTATGAC	Sequencing of UTRs *rhaR*
Ins1cen1	*rhaR*/AN5673	TAACGGAGATGACAGCGATG	Sequencing of UTRs *rhaR*
Ins1cen2	*rhaR*/AN5673	TCGAGTCTTCATAGTCACGAG	Sequencing of UTRs *rhaR*
Ins1cen3	*rhaR*/AN5673	GGTCTCCTCAAGAATATCGAG	Sequencing of UTRs *rhaR*
R0	*rhaR*/AN5673	CGACGCGTGAGGCACAACAGG	Verification of *rhaR* deletion
R1	*rhaR*/AN5673	AGACGCCAGTCAGATACCAGG	Verification of *rhaR* disruption
R2	*rhaR*/AN5673	AGCCTGTCACTACTAGTCC	Verification of *rhaR* disruption
R3	*rhaR*/AN5673	GTGCTAAGCTTCTCGATGG	Verification of *rhaR* disruption; RT-PCR
R5	*rhaR*/AN5673	TAGGGTATTAGGGAGGAAGGG	Verification of *rhaR* disruption
B1	Afu1g13300	CTGGAGGAGTGAAGGATGGGG	Verification of *rhaR disruption*
B2	Afu1g13300	AGAGTTCCGAGGTCGAAGGG	Verification of *rhaR disruption*
N0	*riboB*/AN0670	GGCCTGTAAACCATGCTTGG	Verification of *riboB2::*Af*riboB* strains
N1	*riboB*/AN0670	TCGCTGTGTCATGTACAAGGGG	Verification of *riboB2::*Af*riboB* strains
N2	*riboB*/AN0670	CATTCTATTGCTGTCCGAGCCC	Verification of *riboB2::*Af*riboB* strains
N3	*riboB*/AN0670	TCTCTCTCTGATTGCCCGTTG	Verification of *riboB2::*Af*riboB* strains
F1	Afu1g13300	GTGCGATTACATGAAGGTCC	Verification of *riboB2::*Af*riboB* strains
F2	Afu1g13300	GAGCTGCTTCAACAGACCC	Verification of *riboB2::*Af*riboB* strains
F3	Afu1g13300	ACTTCTTGCATCCGCAGGG	Verification of *riboB2::*Af*riboB* strains
F4	Afu1g13300	ATCCCCTTTCGGTTAGTAACCT	Verification of *riboB2::*Af*riboB* strains
RC6-7	*rhaR/AN5673*	GACGAGAACTGAAGCAGACC	*ΔrhaR* complementation, forward
RC6-8	*rhaR/AN5673*	TGACTTCACCTGGTACCTCC	*ΔrhaR* complementation, reverse
RC6-seq1	*rhaR*/AN5673	GCTTGTTCGTTGGAGACTC	Sequencing of *rhaR*
RC6-seq2	*rhaR*/AN5673	TGGTGTTCGTTATATGTGG	Sequencing of *rhaR*
RC6-seq3	*rhaR*/AN5673	AGCCTGTCACTACTAGTCC	Sequencing of *rhaR*
RC6-seq4	*rhaR*/AN5673	GTGGTTCAATTGGGTCTGG	Sequencing of *rhaR*
RC6-seq5	*rhaR*/AN5673	ACCTACGAAAACGCCTCTC	Sequencing of *rhaR;* RT-PCR
RC6-seq6	*rhaR*/AN5673	GTCACGAATATTGGCGTCC	Sequencing of *rhaR*
Anid-ribo1	*riboB*/AN0670	CATTCTATTGCTGTCCGAGCCC	Amplification and sequencing of *riboB*
Anid-ribo2	*riboB*/AN0670	TCGCTGTGTCATGTACAAGGGG	Amplification and sequencing of *riboB*
nkuA-dir	*nkuA*/AN7753	TCAGCCGGAGAAACGCGTCTCCC	Forward primer to amplify *nkuA*
nkuA-rev	*nkuA*/AN7753	CCAGAACCTCGTGCGCTGAGGGG	Reverse primer to amplify *nkuA*
RC6_SOdir	*rhaR*/AN5673	GAGGATCAAAGTACGAGACCC	Southern probe
RC6_SOrev	Afu1g13300	GTGTCTATGCTGCTGATCTGG	Southern probe
Actin2	*actA*/AN6542	GTACAGATCCTTACGGA	RT-PCR forward
Actin3	*actA*/AN6542	TGTGCAAGGCCGGTTTC	RT-PCR reverse
rhaA35_intron	*rhaA*/AN10277	CAGGCTGGAATAACAACGCC	RT-PCR forward
rhaA_rev	*rhaA*/AN10277	TGGAGCTGCAGTTGAGAGG	RT-PCR reverse
rha122_dir2	*rhaE*/AN7151	CTCTATAGCCACCGCTGATGG	RT-PCR forward
rha122_rev2	*rhaE*/AN7151	TGGAGCTTGTTCAGAAGAGGG	RT-PCR reverse
man_dir	*lraC*/AN5672	AGTTCTCGTCACAATGGCT	RT-PCR forward
man_cen	*lraC*/AN5672	TGAGGAGACCTTCAACACC	RT-PCR reverse
GST:RhaR_Sma	*rhaR*/AN5673	AAATCCCCCCGGGAATGCCGAACGCTGCTATCGCAG	cDNA amplification, forward
GST:RhaR_RI	*rhaR*/AN5673	AACCGGAATTCCATGTGCTGCAGAAGGCTCTGAG	cDNA amplification, reverse
pGEXseq1	pGEX-2T_rhaRcDNA	GGTGATCATGTAACCCATCCT	Sequencing of *rhaR*
pQE80_FOR	pQE80L_rhaRcDNA	CCCGAAAAGTGCCACCTG	Sequencing of *rhaR*
pQE80_REV	pQE80L_rhaRcDNA	GTTCTGAGTCATTACTGG	Sequencing of *rhaR*
^{WT}rhaA-dir1	*rhaA*/AN10277	CTAGCATTACTTCCTTGGGAC	EMSAs
^{WT}rhaA-rev1	*rhaA*/AN10277	TAGAGTTGTTGGATGGCTGGG	EMSAs
^{WT}rhaA R1-dir	*rhaA*/AN10277	AGATCTTCTCGGGGGAAGATCGACCGACCTGGTCC	EMSAs
^{WT}rhaA R1-rev	*rhaA*/AN10277	GGACCAGGTCGGTCGATCTTCCCCCGAGAAGATCT	EMSAs
$^{mut1\ mut2}$rhaA R1-dir	*rhaA*/AN10277	AGATCTTCTC**T**GGGGAAGATCGAC**A**GACCTGGTCC	EMSAs
$^{mut1\ mut2}$rhaA R1-rev	*rhaA*/AN10277	GGACCAGGTC**T**GTCGATCTTCCCC**A**GAGAAGATCT	EMSAs
^{mut1}rhaA R1-dir	*rhaA*/AN10277	AGATCTTCTC**T**GGGGAAGATCGACCGACCTGGTCC	EMSAs
^{mut2}rhaA R1-dir	*rhaA*/AN10277	AGATCTTCTCGGGGGAAGATCGAC**A**GACCTGGTCC	EMSAs
XS22	*xlnA*/AN3613	ATACTCGAGGTACCATAGGATCCTGGAAGTGCG	EMSAs
XN22	*xlnA*/AN3613	GGAATGCATTGTGCTGATCC	EMSAs

[1]Underlined nucleotides are restriction sites added. Capital letters indicate the nucleotides complementary to the gene (gDNA, cDNA, plasmids). Bold letters correspond to the point mutations introduced
