# Supplementary material for: The Aspergillus nidulans Zn(II)2Cys6 transcription factor AN5673/RhaR mediates L-rhamnose utilization and the production of α-L-rhamnosidases
Source: Microb Cell Fact. 2014 Nov 22;13:161. doi: 10.1186/s12934-014-0161-9 (PMC4245848; doi:10.1186/s12934-014-0161-9)
Supplement: Additional file 6: Figure S4 — Construction and gene replacement analysis of the riboflavin nutritional control strains riboB2 ::Af riboB. (A) The correct replacement of AN0670/riboB with the AfriboB expression cassette in the nutritional control complemented strains (AR271-AR273, AR279) was verified using gene specific primers (N1, N2, F1-F4) and primers located outside of the gene replacement cassette (N0, N3). (B) Schematic diagram of the riboB locus and gene replacement events are shown. [file 12934_2014_161_MOESM6_ESM.ppt]

## Slide 1
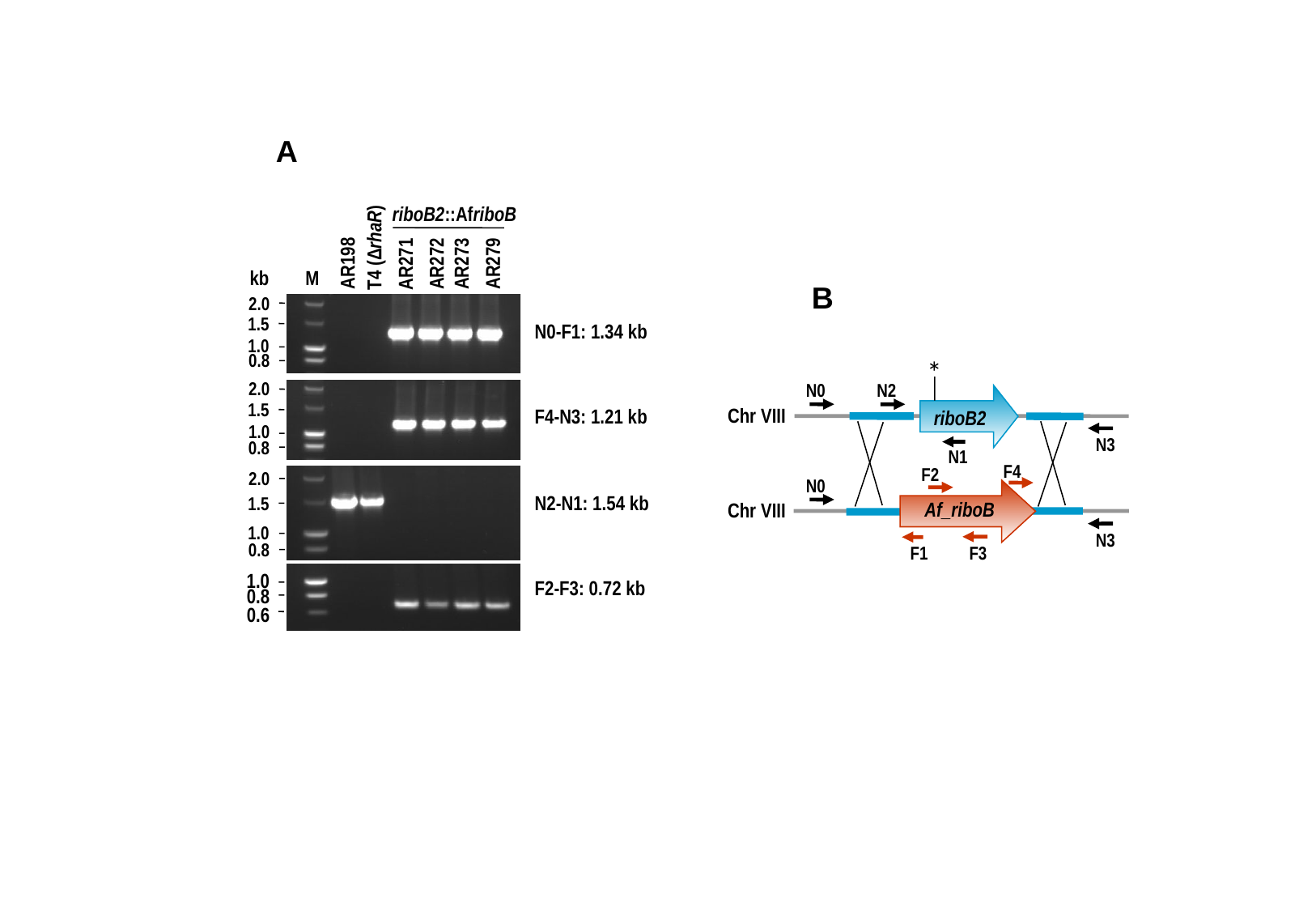

A
T4 (ΔrhaR)
AR198
AR279
AR272
AR273
AR271
kb
M
2.0
1.5
N0-F1: 1.34 kb
1.0
0.8
2.0
1.5
F4-N3: 1.21 kb
1.0
0.8
2.0
N2-N1: 1.54 kb
1.5
1.0
0.8
1.0
F2-F3: 0.72 kb
0.8
0.6
riboB2::AfriboB
B
*
N0
N2
Chr VIII
riboB2
N3
N1
F4
F2
N0
Af_riboB
Chr VIII
N3
F1
F3
